# How *P. aeruginosa* cells with diverse stator composition collectively swarm

**DOI:** 10.1128/mbio.03322-23

**Published:** 2024-03-01

**Authors:** Jaime de Anda, Sherry L. Kuchma, Shanice S. Webster, Arman Boromand, Kimberley A. Lewis, Calvin K. Lee, Maria Contreras, Victor F. Medeiros Pereira, William Schmidt, Deborah A. Hogan, Corey S. O’Hern, George A. O’Toole, Gerard C. L. Wong

**Affiliations:** 1 Department of Bioengineering, University of California Los Angeles, Los Angeles, California, USA; 2 Department of Chemistry and Biochemistry, University of California Los Angeles, Los Angeles, California, USA; 3 Department of Microbiology, Immunology & Molecular Genetics, University of California Los Angeles, Los Angeles, California, USA; 4 California NanoSystems Institute, University of California Los Angeles, Los Angeles, California, USA; 5 Department of Microbiology and Immunology, Geisel School of Medicine at Dartmouth, Hanover, New Hampshire, USA; 6 Department of Mechanical Engineering & Materials Science, Yale University, New Haven, Connecticut, USA; 7 Department of Nanoengineering, University of California San Diego, San Diego, California, USA; Ecole polytechnique federale de Lausanne Global Health Institute, Lausanne, Switzerland; Universite de Geneve, Genève, Switzerland

**Keywords:** swarming, heterogeneous populations, unjamming, stators, flagellar motility, flagellar shut-down, intermittency, crowded environment

## Abstract

Swarming is a macroscopic phenomenon in which surface bacteria organize into a motile population. The flagellar motor that drives swarming in *Pseudomonas aeruginosa* is powered by stators MotAB and MotCD. Deletion of the MotCD stator eliminates swarming, whereas deletion of the MotAB stator enhances swarming. Interestingly, we measured a strongly asymmetric stator availability in the wild-type (WT) strain, with MotAB stators produced at an approximately 40-fold higher level than MotCD stators. However, utilization of MotCD stators in free swimming cells requires higher liquid viscosities, while MotAB stators are readily utilized at low viscosities. Importantly, we find that cells with MotCD stators are ~10× more likely to have an active motor compared to cells uses the MotAB stators. The spectrum of motility intermittency can either cooperatively shut down or promote flagellum motility in WT populations. In *P. aeruginosa*, transition from a static solid-like biofilm to a dynamic liquid-like swarm is not achieved at a single critical value of flagellum torque or stator fraction but is collectively controlled by diverse combinations of flagellum activities and motor intermittencies via dynamic stator utilization. Experimental and computational results indicate that the initiation or arrest of flagellum-driven swarming motility does not occur from individual fitness or motility performance but rather related to concepts from the “jamming transition” in active granular matter.

**IMPORTANCE:**

It is now known that there exist multifactorial influences on swarming motility for *P. aeruginosa*, but it is not clear precisely why stator selection in the flagellum motor is so important. We show differential production and utilization of the stators. Moreover, we find the unanticipated result that the two motor configurations have significantly different motor intermittencies: the fraction of flagellum-active cells in a population on average with MotCD is active ~10× more often than with MotAB. What emerges from this complex landscape of stator utilization and resultant motor output is an intrinsically heterogeneous population of motile cells. We show how consequences of stator recruitment led to swarming motility and how the stators potentially relate to surface sensing circuitry.

## INTRODUCTION

At a macroscopic level, populations of bacteria can abruptly organize into a motile “swarm” (1, 2), but it is not clear how this process is collectively initiated or arrested. The underlying molecular mechanisms that underpin swarming motility are complex. In swarming as in swimming, the flagellar motor provides propulsion (1). The basic flagellar structure is well known, with four main components: the extracellular helical filament, the hook, the rotor, and the stators. When the flagellum is active, the stators transform an ion flux across the cytoplasmic membrane into torque to rotate the rotor. Bacterial species have evolved diverse specialized flagellar motors with differences in (i) the number and type of stators (H^+^ vs Na^+^ powered) (3–5), (ii) the diameter of the C-ring rotor (6), and (iii) presence or absence of a periplasmic ring (7). Even the number and positioning of flagellar motors vary between swarming bacterial species (1, 8). Single stator systems such as that of the multi-flagellated enteric *Escherichia coli* recruit up to 11 H^+^-driven MotAB stators to power its flagella (9, 10), while the marine single-flagellated *Vibrio cholerae* uses up to 13 Na^+^-driven PomAB stators (6). Dual stator systems, such as those for *Shewanella oneidensis* MR-1 (4, 11) and multi-flagellated *Bacillus subtilis* (12), afford these microbes the versatility to utilize two ion gradients by interchangeably recruiting H^+^-driven or Na^+^-driven stators.

How stators are organized with respect to function in *Pseudomonas aeruginosa* is not as clear. *P. aeruginosa* employs a dual H^+^-driven stator system, MotAB and MotCD, but does not exploit different ion gradients, so it is unclear why this apparent stator redundancy exists. In fact, the two stators do not have significantly different torque outputs per stator unit (5). Despite these similarities, the MotAB and MotCD stator sets have been shown to produce remarkably different motility phenotypes. It has been observed previously that a strain with an exclusively MotAB-powered motor can swim faster than its MotCD-powered motor counterpart strain (13, 14), whereas a strain with the MotCD-powered motor forms a significantly larger swarm area than a strain with only the MotAB stator (15, 16), suggesting that these stator sets possess distinct functional capabilities despite their noted similarities. Indeed, it is also not clear how *P. aeruginosa*, a monotrichous species, can swarm so efficiently given that most other bacteria that exhibit swarming motility are either polytrichous species [e.g., *E. coli*, *B. subtilis*, *Salmonella enterica* (1)] or polarly flagellated species that require expression of secondary lateral flagella to swarm [e.g., some *Vibrio* and *Aeromonas* spp. (17–19)].

Here, we examine how *P. aeruginosa* uses different two-stator configurations to initiate or arrest flagellum-driven motility collectively in a population and thereby control swarming behavior. The root phenomenon that enables control of collective flagellum driven motility and environmentally sensitive responses is a biased adaptive stator utilization mechanism. Our data suggest that to facilitate high swim speeds in low-viscosity environments (i.e., swimming), the flagellar motor is primarily decorated with MotAB stators, while high-viscosity environments (i.e., swarming) promote MotCD utilization, which is in turn hindered by high levels of MotAB production. Our data suggest that the torque outputs of motors driven by MotAB or MotCD are not markedly different at high viscosity, consistent with other measurement modalities (5). We find the unanticipated result that the two motor configurations have significantly different motor intermittencies, that is, the fraction of flagellum-active cells in a population on average. What emerges from this complex landscape of stator utilization and resultant motor output is an intrinsically heterogeneous population of motile cells not readily reducible to MotAB-dominant or MotCD-dominant behavior. Based on experimental and computational data, we find that the initiation of flagellum-driven swarming activity occurs when numerous individual motility choices, achieved via stator utilization, are successfully integrated into macroscopic community motion via processes related to the “unjamming” transition in the field of “granular matter,” which separates static, solid-like, sessile behavior from flowing, liquid-like, motile behavior in a population of cells. Conversely, the sudden and collective arrest of flagellum-driven motility can be achieved via changing the composition of stators in the flagellum motor, even for a relatively small subpopulation of cells in a heterogeneous population. The conceptual results presented here are in principle generalizable. Beyond an improved understanding of flagellum-driven swarming phenomena, a collective shutdown of flagellum motility orchestrated by differential stator utilization may be important to surface sensing-mediated signaling pathways that lead to flagellum shutdown during the initiation of biofilm formation.

## RESULTS

### The wild-type (WT) and MotAB motors exhibit greater swimming speed than the MotCD motor in low-viscosity environments, but all strains have similar swimming speed in high-viscosity environments

**Fig 1:**
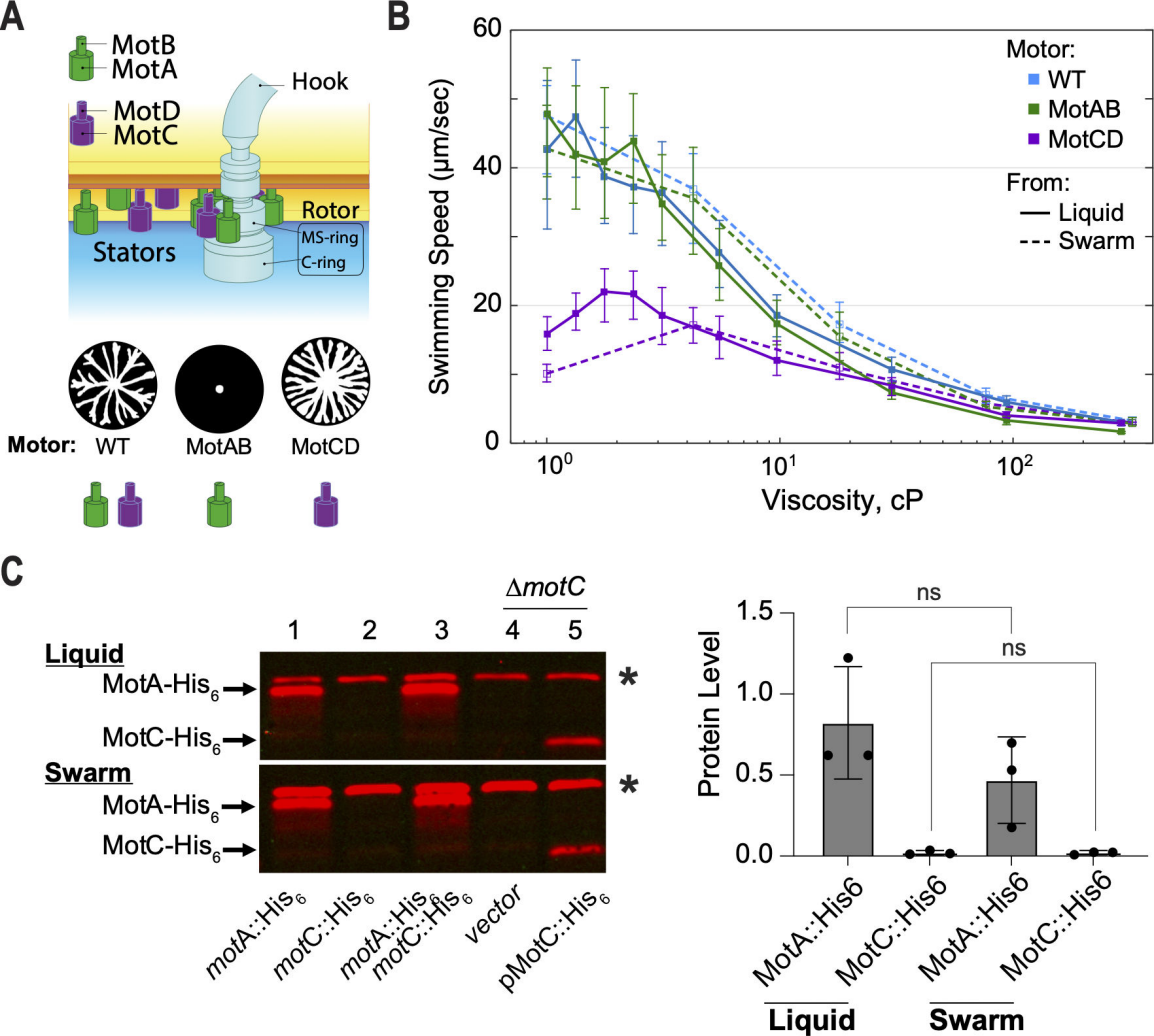
Convergence of swimming speeds by the MotCD motor to the WT and MotAB motors with increasing viscosity and asymmetric production of stator-type sets in WT. (**A**) The dual proton-driven stator system, MotAB and MotCD, of *P. aeruginosa*. Stators are utilized to provide the torque necessary to rotate the flagella. Three motor configurations can be formed: the swarming MotABCD (WT) motor, the swarming deficient MotAB motor, and hyper-swarmer MotCD motor. A representative swarming pattern for each motor type is presented above each stator set. (**B**) Measurement of swimming speeds for the three stator configurations harvested from a liquid planktonic or swarming culture: two-stator WT (MotABCD) and the MotAB and MotCD single-stator motors at different viscosities (by increasing the percent concentration of methylcellulose in solution). At least 100 trajectories per viscosity condition were measured. Error bars denote the first and third quartiles of the distribution about the mean. (**C**) Western blot detection of the MotA::His_6_ and MotC::His_6_ epitope-tagged proteins expressed in membrane fractions of the strains indicated. In lanes 1 through 3, the proteins are expressed from the respective endogenous loci under native promoter control. Samples in lane 3 are derived from the strain in which both the motA and motC genes express the respective proteins fused to the His_6_ epitope. In lanes 4 and 5, samples from the ΔmotC strain harbor either the empty vector control (lane 4) or a multi-copy plasmid for expression of the MotC::His_6_ protein under arabinose induction via the P_bad_ promoter. Arrows point to the location of the indicated proteins. The asterisk (*) indicates a non-specific band present in all samples and used as a normalization control for quantification. Strains were grown for 16 hours in either liquid (top panel) or swarm agar plates (bottom panel) with 0.05% arabinose to induce plasmid-borne MotC::His_6_ expression. Proteins were detected using an anti-His_6_ monoclonal antibody. The bar plot on the right shows the quantification of the MotA::His_6_ and MotC::His_6_ proteins from three biological replicates of samples from the motA::His_6_ motC::His_6_ double-tagged strain grown under the indicated conditions (panel d, lane 3, top and bottom). Data were analyzed by one-way analysis of variance followed by Tukey’s post-test comparison. ns, not significantly different.

A natural starting point of comparison between the output of WT as well as MotAB- and MotCD-exclusive flagellar motors is swimming speeds. Here, it is illuminating to compare single-cell swimming speeds in not only low-viscosity fluids but also high-viscosity environments typically encountered in swarming. Note, that for this manuscript, when we refer to the role of the MotAB motor, we are measuring the behavior of a Δ*motCD* mutant, which lacks MotCD motor function. In contrast, when we refer to a MotCD motor, this shorthand indicates a Δ*motAB* mutant which lacks any MotAB motor function. The WT motor has both functional MotAB and MotCD stators (Fig. 1A).

We find that the WT and MotAB motors facilitated faster swimming than the MotCD motor at low viscosities. In aqueous medium, the linear speed of the MotAB motor (48.5 ± 16.2 µm/s) outperforms that of the MotCD motor by threefold (15.0 ± 5.0 µm/s). However, as viscosity is increased via addition of methylcellulose (20–22), the swim speeds decrease. Importantly, the swim speeds of the three strains converge as extracellular viscosity increases (Fig. 1B). At ~24 cP, the difference in swim speed between the MotAB and MotCD motors is not significant (7.2 ± 3.5 and 8.1 ± 2.9 µm/s, respectively).

Additionally, we tested the effect that the high load swarming environment imposed on the flagella has on swimming speed for the three motor strains. We harvested cells from the edge of the biomass of swarming plate cultures and rapidly introduced the cells to aqueous medium of varying viscosity. We find that swarming environment conditioned cells showed similar swimming speed profiles as those grown in planktonic cultures (Fig. 1B). Only the swarm conditioned MotCD motor showed significant decrease in swimming speeds compared to the planktonic MotCD motor, but this difference is not observed as the viscosity is increased.

Our data indicate that at low viscosity, the MotAB motor is capable of faster swimming than the MotCD motor; however, at high viscosity, both motors perform similarly, a conclusion supported by a recent report (5). Furthermore, generally, no significant differences on swimming speed profiles were observed when the inoculum for this experiment was taken from planktonic vs swarming conditions.

### Production of MotAB and MotCD stators is strongly asymmetric

For the two-stator system of *P. aeruginosa*, the quantity and the type of stator are crucial to understanding motility. While the WT motor is generally thought to utilize MotCD stators to facilitate swarming (23, 24), it is not clear how the MotCD motor strain significantly outperforms the WT and MotAB motor strains in swarming motility. Here, we test the hypothesis that the relative levels of MotAB and MotCD stator pairs in the cell modulate stator composition of the motor.

To answer this question, we measured levels of the MotA and MotC proteins within the same population of cells by inserting a His_6_ epitope tag into the C-termini of the stator proteins encoded by the *motA* and *motC* genes at their respective loci on the chromosome of an otherwise WT strain. This strain allows detection of both the MotA-His (31 kDa) and MotC-His (26.8 kDa) proteins expressed under native promoter control in the same cells via the same antibody but distinguishable by their molecular weight difference. Given that the MotA and MotB subunits are co-expressed in an operon, as are the MotC and MotD subunits, the MotA::His_6_ and MotC::His_6_ protein levels serve as a proxy for the levels of their respective stator pairs, MotAB and MotCD.

As shown in Fig. 1C, levels of the MotA::His_6_ protein (lanes 1 and 3, left panel) and MotC::His_6_ protein (lanes 2 and 3) are not significantly different between liquid and swarm growth conditions (Fig. 1C, right panel). Note that lane 3 samples represent the *motA*::His_6_ *motC*::His_6_ strain bearing both epitope tags. The relative stable levels of MotA and MotC are consistent with numerous expression studies illustrating the unchanging expression levels of *motA* and *motC* genes under different environmental and growth conditions (25–28), as well as with our previous measurement of these protein levels in both WT strains and a non-swarming strain (*ΔbifA* strain) (23).

The MotA::His_6_ protein levels are strikingly higher than those of MotC::His_6_ (Fig. 1C; lane 3 and right panel)—MotC::His_6_ is visually difficult to detect, regardless of whether grown in liquid or on swarm plates. There is approximately 40-fold more MotA::His_6_ protein compared to MotC::His_6_. The low level of MotC is not likely due to destabilization by insertion of the His epitope tag, as we can detect the MotC::His_6_ protein when expressed *in trans* on a multi-copy plasmid (Fig. 1C, lane 5). Furthermore, a strain expressing the MotC-His_6_ epitope tag variant on the chromosome is able to both swim and swarm comparable to the WT strain in plate-based assays (Fig. S1)indicating that the MotC-His_6_ protein is fully functional (compare *motC*::His_6_ images with those for the WT strain). The strains carrying the MotC-His_6_ or MotA-His_6_ MotC-His_6_ epitope tagged proteins also show motility indistinguishable from the WT (Fig. S1), indicating that the epitope tags do not interfere with the function of these proteins.

The measured low levels of MotC compared to MotA may be supported by gene expression regulation. While the *motCD* genes sit in the flagellar gene cluster, regulated by flagellar transcription factors (29), *motAB* operon is instead found in a separate chromosomal location (30), not regulated by the key flagellar factor, FleQ (29). In addition, when we compile expression data from five separate studies on PA, we find that even though *cheAB* and *motCD* sit tandem in the same operon (31), *motC* and *motD* seem to be generally expressed to a lower degree, about 3.2-fold (±0.42) and 3.6-fold (±0.45), respectively(Fig. S2). Their ultimate protein levels may be affected by other factors, such as assembly of functional membrane bound stators and their expected 5:2 MotA:MotB or MotC:MotD stoichiometry.

### All strains exhibit heterogeneous motility populations, each with a characteristic proportion of diffusive to superdiffusive cells

**Fig 2:**
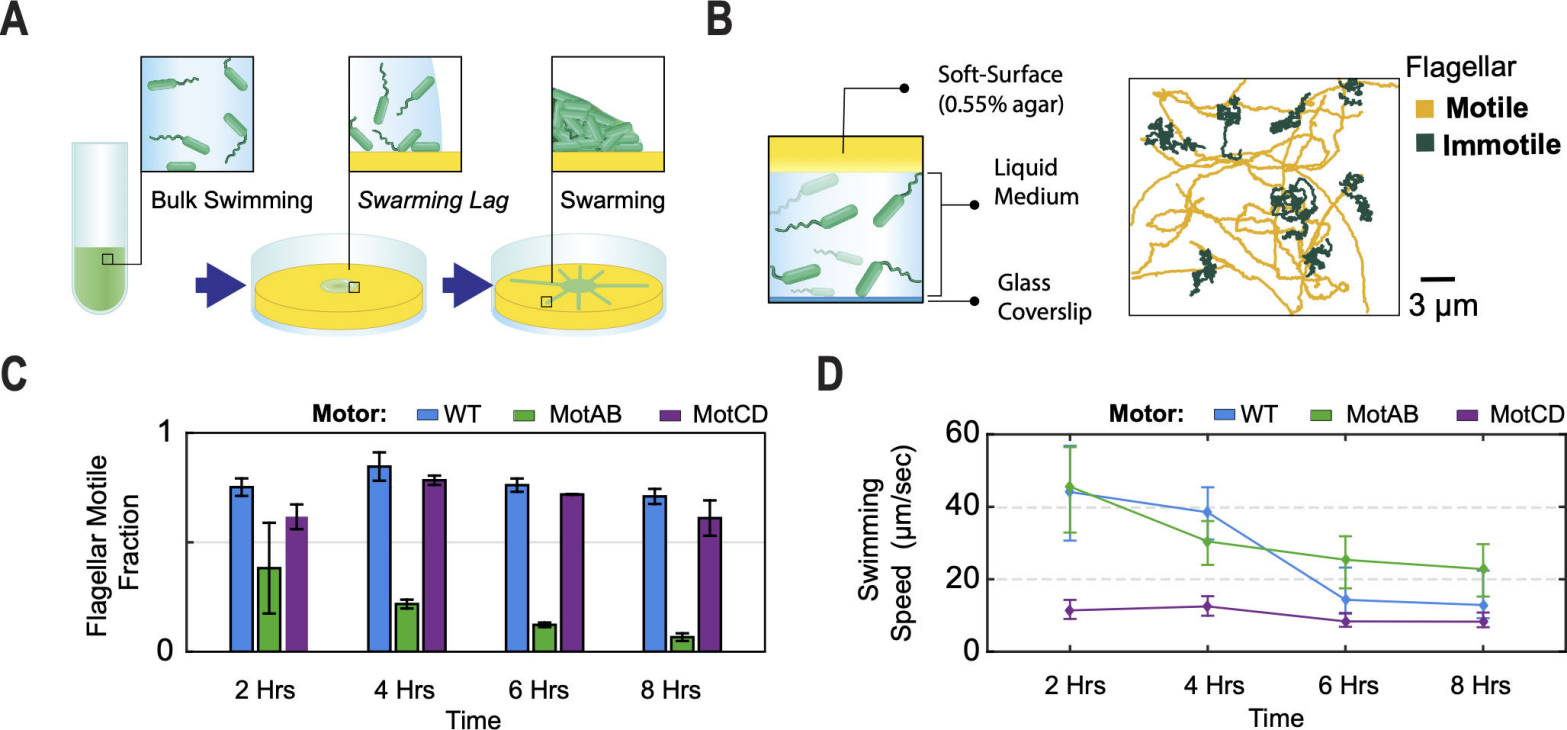
Measurement of fraction of active cells and their swimming speeds in the stagnant liquid agar swarming lag phase environment. (**A**) Diagram of a swarming plate assay experiment. Three stages illustrated: (i) inoculation of cells from liquid growth culture, (ii) a swarming lag phase before cells reach confluency, and (iii) the collective swarming expansion. (**B**) Illustration of experimental setup used for the swarming lag phase microenvironment (left). Two distinct populations were identified in this microenvironment setup (right): flagellar motile cells showed ballistic motion, while flagellar immotile cells moved with a diffusive motion. (**C**) The flagellar motile fraction was quantified using the categories described in **B**. The population activity was tracked every 2 hours for 3 min, over a period of 8 hours. Three replicates were used per strain; at least 140 trajectories were used per timepoint for each replicate. (**D**) Measured swimming speeds performed by the flagellar motile population in plot **C**. At least 200 trajectories per timepoint. Error bars denote the first and third quartiles of the distribution about the mean.

In a typical swarming motility assay, bacteria are transferred from liquid growth to a confined environment consisting of soft (0.55%) agar with a layer of liquid medium. Under these conditions, we find that this system exhibits a delay of about 10 hours before collective swarming expansion (Fig. 2A). This period is similar to the previously observed swarming lag (1). To investigate whether a strain with a MotAB, MotCD, or WT motor shows a different single-cell motility in this pre-swarming period, we prepared miniature soft agar plates using silicone spacers and inoculated a thin layer of liquid cell culture onto its surface (Fig. 2B). The microenvironment was sealed with a glass coverslip, incubated at 37°C, and the motility of single cells was tracked over a period of 8 hours.

We find that all strains show heterogeneously motile populations. We tracked cells and identified two populations with distinctive average motilities: (i) A high motility (defined as “motile”) population exhibiting flagellum-driven ballistic displacement trajectories with mean squared displacement (MSD) slope ≥1.4 from log-log fit (these trajectories are typical for active, propelled motion, rather than diffusive motion, i.e., flagellar motile cells). MSD slope was calculated as defined previously (32). (ii) A low motility (defined as “non-motile”) population exhibiting trajectories that correspond to MSD slope ≥0.7 but ≤1.3 (Fig. 2B, right panel). (By definition, diffusive movement corresponds to an MSD slope of 1, so this “non-motile” population exhibits span a range of motility behavior near the diffusive limit.) Trajectories with MSD slope <0.7 were considered as attached/semi-attached to the glass imaging surface and not used for this analysis. The fraction of flagellar motile cells was significantly larger for the WT and MotCD motor strains compared to the MotAB motor strain—about twofold after 2 hours (75.2%, 61.7%, and 38.4%, respectively). This difference increased to 10-fold after 8 hours primarily due to a large drop in the fraction of motile cells among MotAB motor cells (6.7%). The fraction of motile cells remained relatively constant over time for WT and MotCD motor cells (Fig. 2C), with motile cells as a majority.

Although the MotAB motor strain had a small fraction of motile cells, their swimming speed was faster than the strain with the MotCD motor (45.6 ± 10.6 vs 11.4 ± 2.9 μm/s at *t* = 2 hours; Fig. 2D). The MotAB motor strain progressively decreased its speed over time to 22.8 ± 7.3 μm/s after 8 hours of incubation but remained significantly faster than the MotCD motor strain (8.3 ± 2.0 μm/s). The WT motor started with a median speed of 44.1 ± 13.1 μm/s after 2 hours, comparable to the MotAB motor strain, and it slowed down to 12.9 ± 6.6 μm/s after 8 hours, closer to the MotCD motor strain speeds. The relatively constant diffusivity of the immotile cells indicates that the slowdown in swimming speeds across the strains over time is not due to an increase in viscosity under our experimental conditions (Fig. S3).

### Single-cell tracking of swarming cells reveals that MotAB and MotCD motors result in drastically different long-term intermittency in flagellar activity

**Fig 3:**
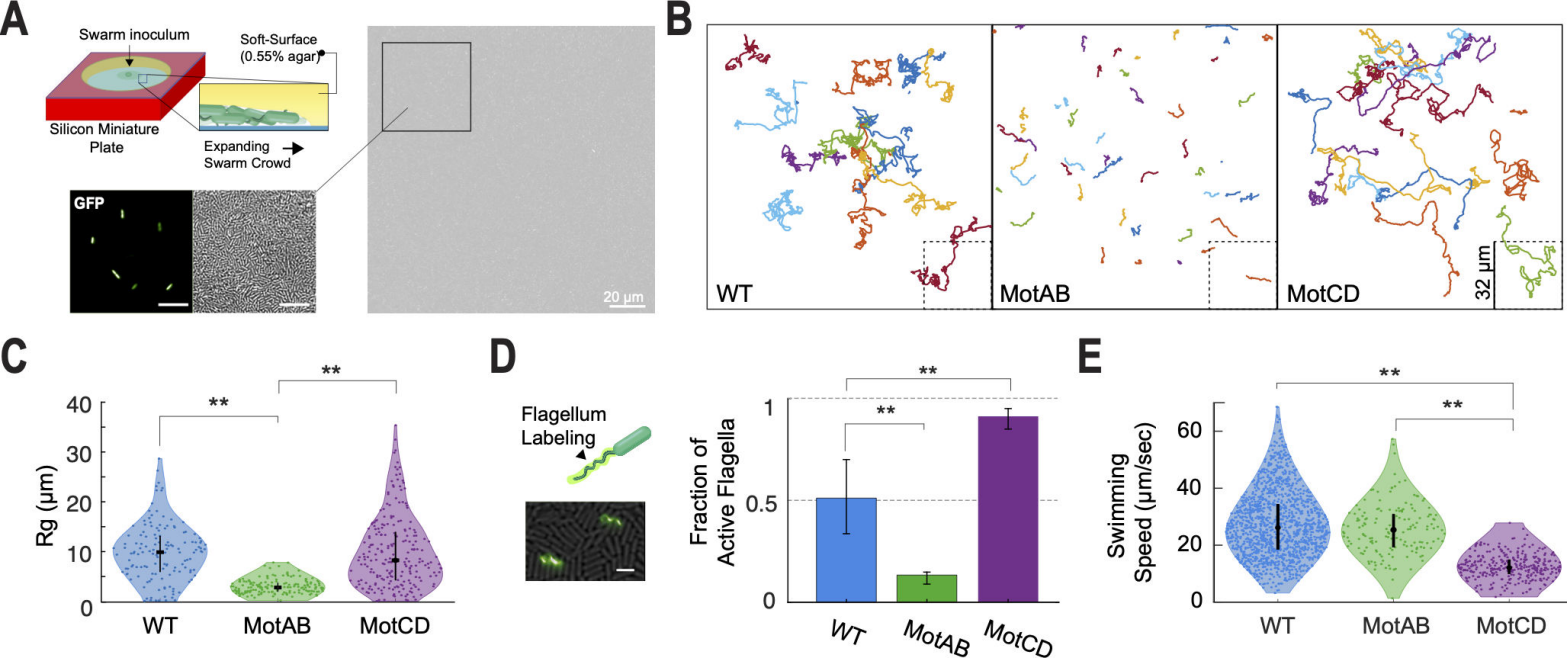
Single-cell motility measurement of cells in a crowded environment of their own kin and 2D confinement. (**A**) Diagram of experimental setup for tracking swarming bacteria on a soft agar medium surface (top left). Cells harvested from a swarm plate with 1%–5% (vol/vol) co-culture of cells carrying a constitutively expressed copy of GFP on a plasmid (lower left, right). This approach permitted precise single-cell tracking in an environment crowded with thousands of cells per field of view. Scale bar in zoom-in inserts, 10 µm. (**B**) Representative trajectories of tracked cells in the crowded environment over a period of 30 seconds. The trajectories presented for each indicated strain come from a compilation of different fields of view from at least three replicates. (**C**) Violin plot of measured radius of gyration for the single-cell trajectories in the crowded environment for the three indicated strains, as displayed in **B**. At least 139 cells were tracked per strain. (**D**) Co-inoculation containing a small fraction of cells, 5%–20% (vol/vol), with a FliC^T394C^ mutation for maleimide staining, was used for direct quantification of actively rotating flagella. The bar plot on the right reports the fraction of active flagella observed for each strain. At least 12 fields of view from four replicates were used and at least 200 flagella per strain were counted. Scale bar, 5 µm. (**E**) Swimming speeds of cells moving in a 2D thin liquid volume medium confined between a 0.55% agar surface and imaging glass coverslip, under a diluted cell volume fraction, Φ (Movie S4). At least 180 trajectories were measured per strain. For panels C–E, data sets were analyzed by one-way analysis of variance followed by Tukey’s post-test comparison. *P  <  0.05, **P < 0.001; ns, not significantly different.

To trace the origins of collective swarming motility by *P. aeruginosa*, it is important to not just measure population averaged behavior but also determine the single-cell behavior that may contribute to this collective behavior. To assess contributions of strains using one vs both stators during swarming motility, we tracked single cells from the edge of an early swarm—just as the tendrils begin to form. Cells harvested from the edge of the swarm were inoculated to the center of a miniature 0.55% agar plate (Fig. 3A). To track single cells in the crowded swarm environment, the initial swarming plates were inoculated with a co-culture containing a tracer fraction of cells carrying a constitutive green fluorescence protein (GFP) plasmid (Fig. 3A) at 1%–5% (vol/vol) of GFP cells.

In this crowded condition, the MotAB motor strain showed significantly shorter translational displacement compared to WT and MotCD motor strains (Fig. 3B). Such differences are quantitatively reflected by the radius of gyration (Rg) of the trajectories, which describes a characteristic spatial extent of cell travel at the longest observation time (33). The Rg is defined as:


$${R_{g}}^{2}=\frac{1}{N}\sum_{i=1}^{N}(\vec{R_{i}}-\vec{R_{cm}}{)}^{2},$$

where *N* is the number of points in the track, $\vec{R_{i}}$ is the position vector of the *i^th^* point on the trajectory and is the center-of-mass $\vec{R_{cm}}$ of all points. The WT and MotCD motor strains display trajectories up to 10 times longer than the mean trajectories of the MotAB motor strain (Rg = 6.2 ± 3.8, 6.1 ± 4.5, and 1.9 ± 0.9 µm, respectively; Fig. 3C).

To quantify the flagellar activity that drives the motility behaviors observed under the crowded conditions described above, we harvested cells from the edge of an early swarm from a co-culture containing a tracer population of cells carrying a threonine-to-cysteine mutation in the flagellum filament subunit (FliC^T394C^) (34), analogous to the single-cell tracking approach described above in Fig. 3A (with 5%–20%, vol/vol, of FliC^T394C^ cells). The flagella were stained with an Alexa Fluor 488 C5 maleimide added to the plate prior to harvesting (Fig. 3D). This approach allowed for direct observation of the fraction of active flagella. While almost all the flagellated cells using the MotCD motor were active (92% ± 6%), only 13% ± 9% of the MotAB motor strain had an active flagellum (Fig. 3D; Movies S1; S2). The WT motor had an intermediate fraction of active flagella, but also with the largest variation, 51% ± 24% (Movie S3). The percentage of active flagella observed here aligns with the differential percentage of motile cells measured above during swarming lag.

Finally, we characterize the flagellar output under this confined monolayer geometry. Since in the crowded regime, it is not possible to untangle the flagellar output from the contributions of all neighboring particles to the movement of a single cell, we diluted the system to measure the free-swimming speeds of cells in a 2D confined (monolayer) liquid volume. Cells harvested from the edge of a swarming motility plate were diluted and inoculated as a thin liquid film, ~1.5 µm in height, between a 0.55% agar surface and an imaging cover glass (Movie S4). In this free-swimming configuration, the WT and MotAB motors outperform the MotCD motor by about twofold (26.2 ± 11.5, 25.4 ± 8.8 and 12.1 ± 4.2 µm/s, respectively). Hence, the agar-liquid-glass monolayer configuration imposes a characteristic load on the flagellum equivalent to a moderate viscosity, i.e., ~10 cP (Fig. 1B). Therefore, under this 2D monolayer configuration, as crowding increases (Fig. 3A), we expect such flagellar load to increase due to cell-to-cell interactions (collisions) and lead to a convergence in motor output between the strains like the measured behavior above with increasing flagellar viscosity load.

### Modeling of cell populations with heterogeneous motor output reveals the existence of unanticipated unjamming transition modalities

**Fig 4:**
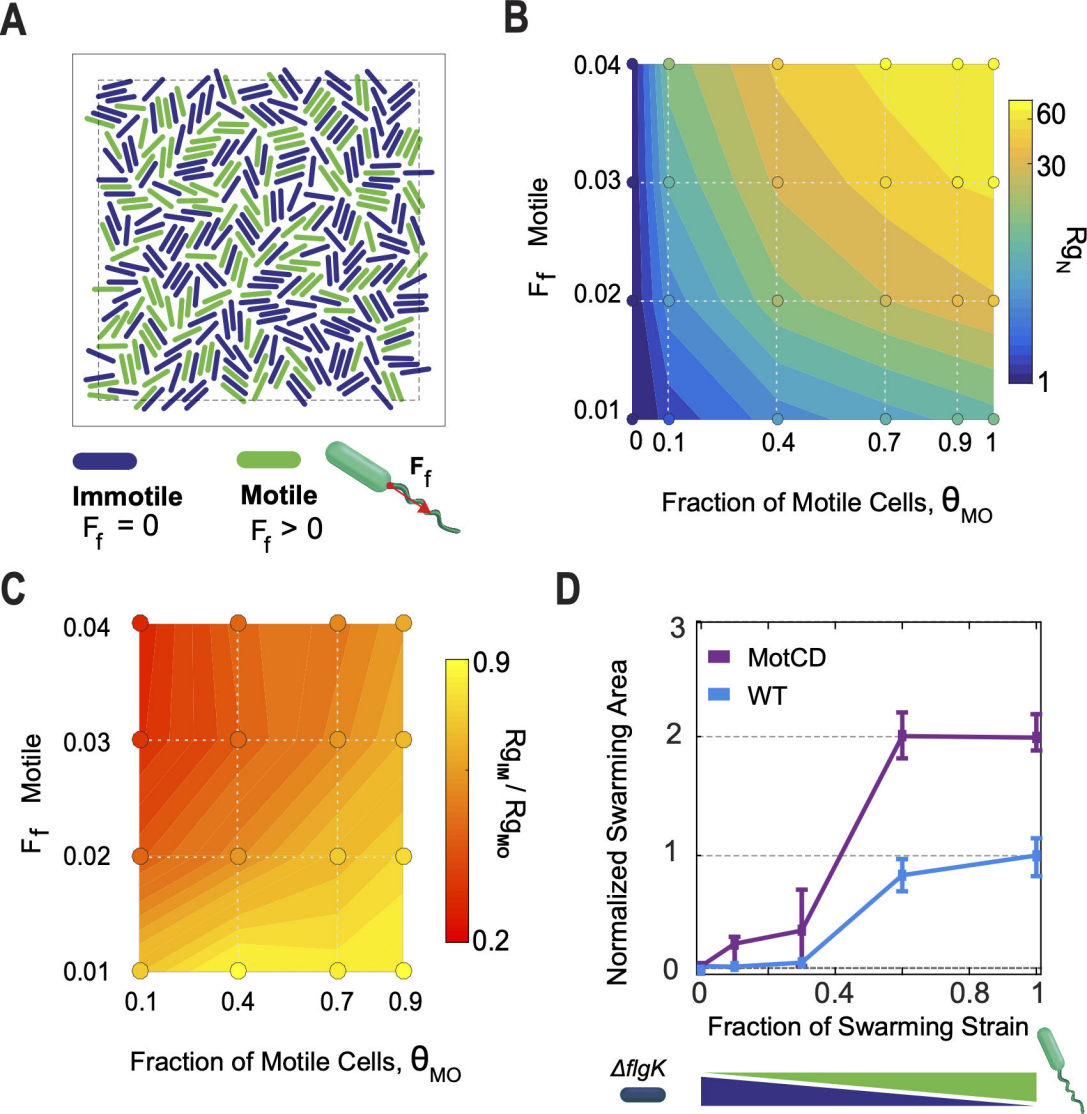
Physical modeling of the crowded environment predicts a landscape of unjamming transitions for different combinations of the flagellum motor force output and fraction of active flagellum cells. (**A**) Simulations of a crowd of self-propelled rods to evaluate the influence of population heterogeneity, flagellar motile vs immotile, with varying flagellar force outputs on collective motility. The simulations were tested at a volume fraction (Φ) of 0.96 and cell aspect ratio of 4. The fraction of motile particles (θ_MO_) and their flagellar output (*F*_*f*_) were varied. (**B**) Normalized mean radius of gyration, Rg_N_, for the particles in the tested crowded systems as illustrated in **A**. The values were normalized by the mean radius of gyration of the homogeneous system with all immotile particles (*F*_*f*_ = 0). Contour map was estimated by interpolation between the grid of tested conditions (circular markers). (**C**) The asymmetry in translational movement between the motile and immotile fractions for the heterogeneous systems was measured as the ratio in mean Rg for the two populations (Rg_IM_/Rg_MO_). An Rg_IM_/Rg_MO_ < 1 corresponds to longer trajectories performed by the motile fraction, relative to the motility that the motile fraction induced on the immotile fraction in the crowd; a ratio of 1 corresponds to equal degree of translation performed by both immotile and motile particles. (**D**) Normalized swarming area as a function of increased concentration of the swarming strain (WT and MotCD motors) in mixed culture with the flagellum-deficient ΔflgK mutant. The ΔflgK strain lacks a functional flagellum and, hence, is swarming deficient. Error bars denote the quartiles of the distribution about the mean.

To examine how diverse flagellar motor outputs and intermittency (i.e., fraction of motile cells) in a heterogeneous population are integrated into either a collective swarming or non-swarming phenotype, we designed a physical simulation model of self-propelled rods (aspect ratio = 4) in a 2D crowded environment with a volume fraction, Φ, of 0.96. These are conditions akin to the high-density environment of swarming cells. Each simulation contained a fixed fraction of immotile (*F*_*f*_ = 0) and motile cells (*F*_*f*_ > 0) (Fig. 4A; Movies S5–S8); hence, each system contained a characteristic fraction of flagellum active cells, θ_Mo_. The relative magnitude of the active force is reported compared to the repulsive interaction coefficient, *k*, of the repulsive linear spring potential for the particles (see SI Appendix). To evaluate the extent of movement within the crowd, we report here the collective radius of gyration, Rg, for the systems normalized by the radius of gyration of the homogeneous system with all immotile particles and denoted as Rg_N_ (Fig. 4B).

The progressive increase in fraction of motile cells promotes incremental movement via a dynamical and cooperative transition analogous to an unjamming transition in the field of active fluids (35–38). In homogeneous motile systems, θ_Mo_ = 1, the translation is positively dependent on the flagellar force (Fig. 4B). Interestingly, the data also show that it is possible to achieve comparable large displacements under multiple configurations in the heterogeneous systems. For instance, the system with 0.9 θ_Mo_ fraction and *F*_*f*_ = 0.02 promoted similar crowd movement to a system with 0.4 θ_Mo_ and *F*_*f*_ = 0.03 (37.8 and 31.8 Rg_N_, respectively).

While Rg_N_ reflects the mean motility in the heterogeneous systems, the motility distribution induced by the changing motile fraction upon the entire system is multimodal. Interestingly, the mean Rg of the immotile cells (Rg_IM_) has a complex relationship with that of the motile cells (Rg_MO_) in the crowded system, which implies interplay between the two populations in this regime. These effects can be seen in the mean Rg ratio between the two population fractions, Rg_IM_/Rg_MO_. Such ratios are not dictated exclusively by the fraction of motile cells but similarly depend on the force (Fig. 4C). For example, both, a system with 0.9 θ_Mo_ fraction and *F*_*f*_ = 0.01 and one with 0.1 θ_Mo_ fraction and *F*_*f*_ = 0.03, can induce a similar Rg_N_ in the crowd; however, the former system has greater symmetry in the translational motility performed by the motile and immotile fraction (Rg_IM_/Rg_MO_ = 0.87), i.e., motile fraction induced equivalent motility to their own onto immotile fraction, compared to the latter system (Rg_IM_/Rg_MO_ = 0.26). A lower Rg_IM_/Rg_MO_ ratio is relevant to the behavior that has been observed in heterogeneous mixtures of hyper-swarming and swarming strains (39) or species (40), in which the hyper-swarming cells move through the swarm to lead the swarming front.

Since viscosity plays an important role in bacterial swarming, we tested how changes in translational and rotational frictional forces acting on each particle, Eq. 3, 5, and 7, affected the motility features presented above. Although the systems are slowed down as expected, the dynamic landscape of compensating contributions of θ_M_ and *F*_*f*_ is conserved across viscosities (Fig. S4A-D). In comparison, the shape of the Rg_IM_/Rg_MO_(θ_M_, *F*_*f*_) map is generally conserved, but favoring higher values of Rg_IM_/Rg_MO_ with increasing viscosity (Fig. S4E-H). Furthermore, slowdown of the systems due to increments in viscosity favors higher MSD log-log slopes at late lag times (Fig. S4I-L), contributing to enhanced collective diffusivity. The increase in diffusivity, however, is not unform; instead, we find that specific configurations of the θ_M_ and *F*_*f*_ parameters create a diffusivity “sweet spot” for the different viscosities modeled, favoring higher values of θ_M_ and *F*_*f*_ as viscosity increases.

### Modulation of swarming motility in *P. aeruginosa* via modulation of flagellum-active populations

Based on the observed characteristic dynamic distribution of inactive cells between the WT, MotAB, and MotCD motor strains and our model estimations on how such active to inactive ratios will impact the promotion or arrest of swarming motility, we predict that altering the proportion of inactive cells in a population of swarm-competent cells would impact swarming behavior at a macroscopic level. To test if and how the fraction of inactive cells impacts swarming, we controlled the fraction of active cells by mixing the swarming strains (WT and MotCD motors) with a flagellum-less strain (Δ*flgK*) at different static ratios, similar to an approach we used previously (41). As shown in Fig. 4D, swarming motility of both the WT and MotCD motor strains is negatively impacted by increasing the proportion of inactive Δ*flgK* cells in the population, consistent with our prediction and our previous report (41). For the MotCD strain, swarming onset occurs when MotCD cells comprise ~0.1 to 0.3 fraction of the mixed population. To calculate the fraction of cells with active flagella in this mixed population, we must consider our results above (Fig. 3D), whereby the WT and MotCD swarming strains exhibit distinct proportions of flagellum-active and flagellum-inactive cells in their populations. Taking such data into account, the fraction of the MotCD strain with active flagella at the onset of swarming in the MotCD/Δ*flgK* mixed population is ~0.18 (i.e., 0.9 fraction of active cells in the MotCD strain alone and an average of 0.2 fraction of the mixed MotCD/Δ*flgK* population at swarming onset). For the WT strain, the fraction of the WT population with active flagella at the onset of swarming is ~0.15 (with 0.5 fraction active cells in WT and 0.3 fraction of the mixed WT/Δ*flgK* population). Notably, both values are comparable to the measured fraction of flagellum-active cells of the swarming-deficient strain, MotAB motor (~13%), indicating that increasing the fraction of inactive cells in WT or MotCD motor swarming populations can effectively mimic the lack of swarming observed for the MotAB motor strain.

### Pro-flagellar shutdown effect of MotAB stator in WT can be offset by an increase in MotCD production

**Fig 5:**
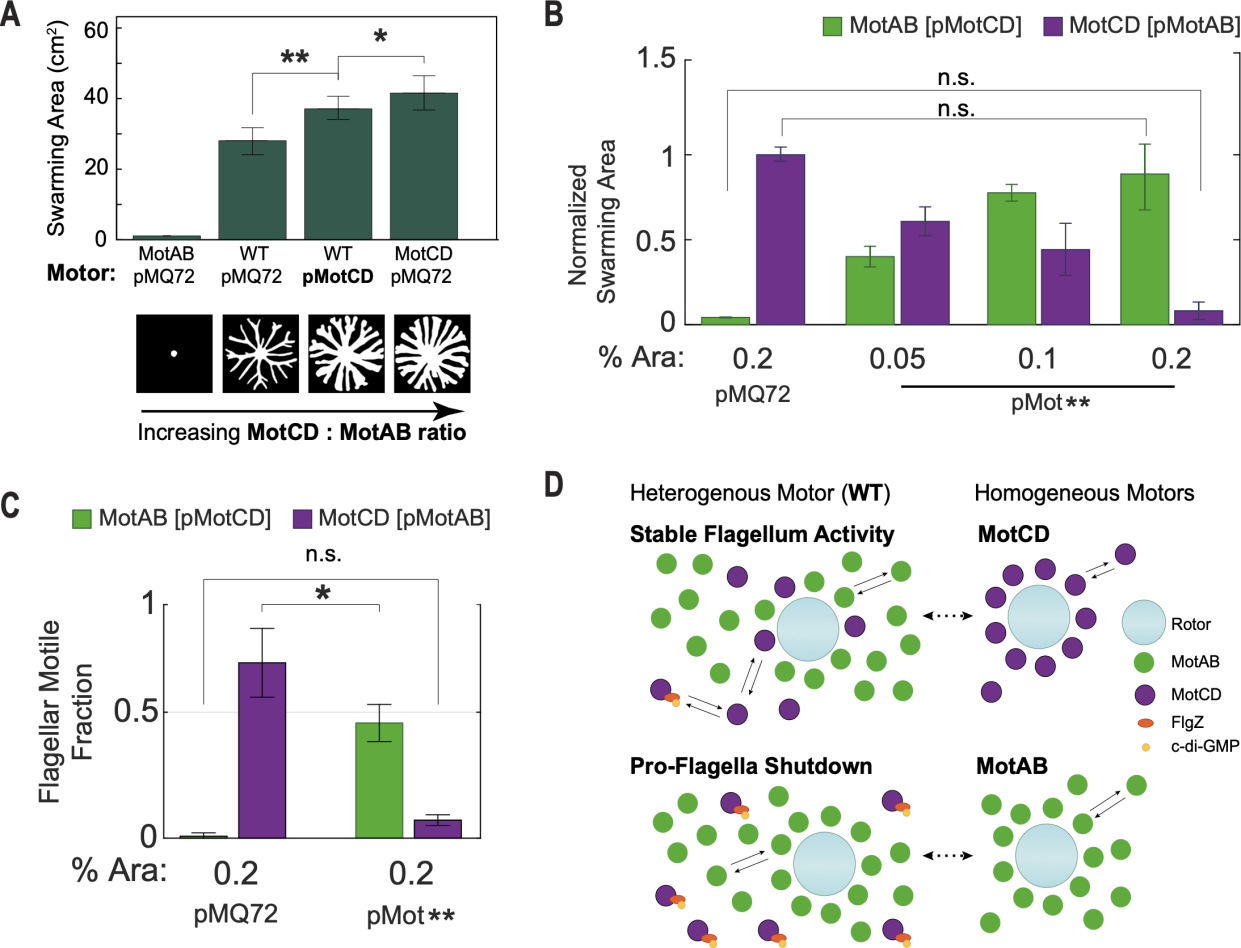
Increasing MotCD:MotAB ratio leads to increased swarming motility. (**A**) Expression of MotCD via an arabinose-inducible plasmid (pMotCD) increases WT swarming phenotype (pMQ72 is an empty vector control). All strains were grown in soft agar swarming plates with 1% arabinose. No significant difference was observed between the WT/pMQ72 and WT/pMotCD at 0% arabinose. At least six plate replicates per condition. Error bars denote the first and third quartiles of the distribution about the mean. (**B**) Alternative expression via arabinose-inducible plasmid of MotCD (pMotCD) or MotAB (pMotAB) in the MotAB motor or MotCD motor strain, respectively. Progressive expression of pMotCD induced swarming motility in non-swarming MotAB motor, even to comparable levels to MotCD motor strain swarming; inversely, increasing induction of pMotAB leads to a progressive swarming arrest of hyper-swarming MotCD motor strain. (**C**) Quantification of flagellar motile fraction at 0.2% arabinose induction of pMotCD or pMotAB in the MotAB motor or MotCD motor strain, respectively. pMotCD increased fraction of motile cells in the MotAB motor strain, while pMotAB reduced the flagellar motile fraction in the MotCD motor strain. *P  <  0.05, **P < 0.001; ns, not significantly different. Data were analyzed by one-way analysis of variance followed by Tukey’s post-test comparison. Lower panel shows representative segmented swarm areas for the four different stator ratio configurations. (**D**) Model of stator-type dynamics and their expected influence on the motor intermittency in a crowded swarming environment (high flagellar load). All three motor types are expected to maximize flagellar output under this viscous condition, i.e., motors fully or mostly decorated with stators. The WT heterogeneous motor asymmetrically uses MotAB stators due to its higher affinity at low-to-mid range viscosities and elevated expression compared to the MotCD stator (Fig. 1B and C). To further hinder the motor utilization of MotCD, we previously described the cyclic di-GMP-dependent binding of FlgZ to the MotC subunit of this stator and its sequestering from the motor (15, 42). We postulate here that the presence of MotCD stators helps stabilize the flagellum activity, i.e., maintaining an active flagellar motor. The MotCD motor sustains populations a high flagellar motile fraction. In contrast, the MotAB motor is prone to a flagellar shutdown with an extremely low flagellar motile fraction. We propose that the rivaling influence of MotAB and MotCD on the flagellar motor is dependent on the relative levels of “free” available stators between the two stator types, with such availabilities being influenced by stator production and molecular interactions. Hence, as well as modulating the flagellar torque output, the MotAB and MotCD stators may integrate molecular signals to regulate the flagellar activity state, long-term intermittency, among the heterogeneous population.

Given that expression of MotAB dominates that of MotCD, we hypothesize that the MotAB stator can be utilized in the WT motor during swarming, thereby hampering a heterogeneous motor from reaching higher degrees of swarming, e.g., MotCD motor-like hyper-swarming. A strong prediction of this model is that a MotCD-dominated motor would enhance swarming motility. This prediction aligns well with our experimental data shown in Fig. 4D. We previously showed that deleting the MotAB stator results in a hyper-swarming phenotype (15, 23). Here, we utilized a plasmid carrying the *motCD* genes, whose expression is under arabinose-inducible control, to increase expression of MotCD in a WT background. We observed an arabinose-dependent increase of swarming motility with increased expression of MotCD (Fig. 5A). At 0% arabinose, the swarming motility between the WT pMotCD strain and the WT carrying the empty vector (pMQ72) was not significantly different (Fig. S5). When induced, MotCD expression (1% arabinose) led to a mean increase of ~40% in swarming area compared to the WT pMQ72. Interestingly, we stress that this increase in swarming motility did not result in an increase in swimming speeds (Fig. S6). We attribute this to the expected MotAB preference for motor utilization in low-viscosity planktonic motility (Fig. 1B).

To demonstrate in a different way the contrasting effects that MotAB and MotCD have on setting the population flagellar intermittencies, we alternatively introduced an arabinose-controlled plasmid carrying one stator into the motor strain lacking said stator, i.e., the MotAB stator into the MotCD motor strain and the MotCD stator into the MotAB motor strain. The plasmid expressions had similar effects to the ones observed in WT, but the contrasting swarming phenotypes of the MotAB and MotCD motor strains allowed for a more gradual effect on swarming phenotypes as stator availability increased via expression (Fig. 5B). Controlled expression of the MotAB stator progressively reduced the swarming phenotype of the hyper-swarming MotCD motor strain background, eventually leading to swarming arrest at the highest arabinose concentration tested (0.2% Ara). Furthermore, progressively increasing expression of the MotCD stator in the non-swarming MotAB motor strain background led to a gradual increase in swarming, ultimately reaching swarming motility comparable to that of the hyper-swarmer MotCD motor strain. Consistent with our hypothesis of the influence of stators on the motile fraction (or immotile fraction) of a population, overexpression of MotAB significantly reduced the fraction of motile cells in the MotCD motor strain from 0.70 ± 0.14 (pMQ72) to 0.07 ± 0.02 at 0.2% Ara induction of MotAB plasmid, while overexpression of the MotCD stator significantly increased the fraction of motile cells in the MotAB motor strain from 0.01 ± 0.01 (pMQ72) to 0.46 ± 0.07 at 0.2% Ara induction (Fig. 5C).

Note that as with WT, plasmid expression of MotCD in the MotAB motor strain leads to a swarming motility increase, analogous to the MotCD motor strain, but did not result in a full recovery of the MotCD motor strain hyper-swarming phenotype. This difference in motility between the MotCD motor strain and the MotCD plasmid expressing strains may be attributed to the non-zero presence of MotAB in the WT and MotAB motor strains. In fact, consistent with our model, the fraction of motile cells in the MotCD motor strain is higher than that in the MotAB motor strain expressing MotCD at 0.2% Ara (Fig. 5C).

## DISCUSSION

In this work, we propose a conceptual framework through which the collective flagellum-driven motility in a population of *P. aeruginosa* cells can be controlled via dynamic MotAB/MotCD stator utilization. An important unanticipated observation presented here from direct measurement of single-cell flagella tracking in a crowd of swarming cells is that MotAB and MotCD motors have drastically different intermittency in flagellum activity (~12% MotAB vs ~92% of MotCD motors are flagellum active) (Fig. 3D). This finding implies that stator utilization can strongly impact the fraction of time that a cell’s flagellum is on. Based on our experimental and modeling data, we propose that swarming in *P. aeruginosa* occurs when numerous individual motility choices achieved via stator utilization are successfully integrated into macroscopic community motion via processes related to “unjamming,” which separates a static solid-like biofilm from a flowing liquid-like swarm in “granular matter.” In a more general compass, these results suggest that deployment of heterogeneity in a motile microbial population can drive unexpected collective motility behavior.

Swarming has traditionally been described by necessary and sufficient molecular component requirements that are deterministic in nature (e.g., the appropriate stators). The data here indicate that this perspective is too reductionist. We find that populations with either MotAB, MotCD, or WT motors all exhibit heterogeneous motility populations, each with distinct characteristic proportions of highly motile superdiffusive cells (Fig. 2C and D). Instead of a conceptual framework where MotAB contributes less to swarming than MotCD, we find that MotAB and MotCD have, in fact, complex and sometimes complementary contributions to flagellar activity (or inactivity) during swarming (Fig. 5B), with MotCD maintaining a population that is motile on average and MotAB instead maintaining a population of individual cells that contribute infrequent spasms of motility (Fig. 5D). We propose that this effect of MotAB-induced flagellum intermittency may be the first step in a full flagellum shutdown in high-viscosity environments, i.e., high flagellar load. Indeed, our proposed picture is not inconsistent with previous experimental observations in which only ~3.5% of flagellum-tethered MotAB motors spin (13). We find that a MotAB motor is especially prone to shut down due to intrinsic intermittent activity, which we observe even in cells not associated with the surface.

The concept of a *P. aeruginosa* population that is intrinsically heterogeneous with different flagellum activity levels, due to its adaptive stator utilization, allows us to engage a new perspective on heterogeneity by importing concepts from “active granular matter” (43–46). We have shown that a population with heterogeneous motility can integrate into a swarm via different combinations of motile cell fractions and flagellum motor force output (Fig. 4B). We find that a sliding scale of different combinations of these parameters can all achieve community motility. Although increasing the fraction of flagellum active cells is a requirement for increased mobility of crowded systems, a majority of flagellum active cells are not necessary. Even when the flagellum-inactive cells are the dominant population, the system may be able to maintain motility, as seen in the WT and MotCD motor strain swarming inhibition experiments (Fig. 3D), as well as recent studies by Hogan et al. (41) and Xavier et al. (2, 47). That is, even cells that are slower or not actively pushing can, counterintuitively, promote swarming of the population by effectively creating local free volume and allowing flow. We find that such features of compensating flagellum force vs population intermittency are conserved at different simulated viscosities (Fig. S4A-D). While the net effect of increasing viscosity is to slowdown the system, viscosity seems to have a positive effect on key features of the collective motility, such as increasing the level of displacement of immotile cells to be on par to that of the motile fraction (higher Rg_IM_/Rg_MO_) and increasing the system’s MSD slope at late lag times toward a more directional collective diffusion (Fig. S4E-L). It is interesting to note that enhancement of collective motility by slow displacements has been observed in the collective pili-mediated twitching motility of *P. aeruginosa*, a completely different motility mechanism (48).

We note that different regulators of motility, such as cell-cell pili interactions (41, 49), secretion of extracellular polymeric substances (41, 50), and secretion of rhamnolipids (47), have all been observed to impact swarming. However, from the present perspective, it is interesting to see how these factors relate to sensing events that may parallel swarming. For example, how do bacteria sense local effective viscosities or the “crowdedness” of local environments? These molecular factors alone can affect the rheology of the media that the bacteria encounters (51–53). There are numerous examples in the colloidal suspension literature (45, 54) showing that the effective viscosity that the suspended particles experience increases dramatically relative to the nominal viscosity of the solvent as the packing fraction of the colloids increases toward the onset of jamming (46). Since the two stator types have different viscosity sensitivity for motor recruitment (5) and utilization (our data), the question of how *P. aeruginosa* measures viscosity or crowding is a salient one.

The MotAB/MotCD competition for motor utilization is not exclusively controlled by production and viscosity. It is also affected by the stators protein-protein interactions. Previously, we showed that the PilZ domain-containing protein FlgZ binds to MotC in a c-di-GMP-dependent manner which can prevent the MotCD stator from integrating into the flagellum motor and hence reduce MotCD availability to the motor (15, 42). Interestingly, we previously showed that MotC can interact directly with the diguanylate cyclase SadC leading to an increase in activity of SadC, i.e., an increase of c-di-GMP levels in the cell (15). Consequently, MotCD stators not recruited to the flagellar motor may influence its own recruitment. Indirectly, MotAB can also participate in such c-di-GMP-related MotCD interactions by preventing MotCD recruitment into the motor by its own occupancy. Such a motor state combined with heavy flagellar loads may render the resulting MotAB motor effectively inactive, since this stator is prone to infrequent flagellum activity. In fact, plasmid overexpression of MotAB in WT not only leads to swarming arrest but also to an increase of c-di-GMP (15). In contrast, plasmid-controlled MotCD overexpression in the WT background results in swarming enhancement (Fig. 5A), suggesting an increase in the motile fraction of the biomass. The effects of MotAB and MotCD in the flagellar heterogenicity of the swarm are key contributors to regulation of the degree of swarming (Fig. 5B and C). We propose that the relative levels of “free” MotAB and MotCD exert a strong influence on the fractions of immotile cells and motile cells.

The conceptual results presented here are critical for understanding swarming. However, the idea of stators that lead to drastically different motor intermittencies is generalizable to bacterial sensing phenomena. For example, a collective shutdown of flagellum motility orchestrated by stator recruitment may be important to surface sensing-mediated signaling pathways that lead to flagellum shutdown and nucleation of microcolonies in heterogeneous bacterial populations for biofilm formation.

## MATERIALS AND METHODS

### List of strains

The following plasmids were used in the construsction of the strains listed in Table 1 for this study: pSMC21 constitutively expresses *gfp* and confers resistance to kanamycin (200 µg/mL), pMQ72 allows for inducible expression by addition of arabinose and confers resistance to gentamycin (25 µg/mL).

**TABLE 1:** List of strains

Strain #	Genotype	Source
SMC232	*P. aeruginosa* PA14	Rahme 1995 (55)
SMC5333	*P. aeruginosa* PA14 *ΔmotCD*	Kuchma 2015 (23)
SMC5769	*P. aeruginosa* PA14 *ΔmotAB*	Kuchma 2015 (23)
SMC6709	*P. aeruginosa* PA14 *fliC*^*T394C*^	de Anda 2017 (34)
SMC7580	*P. aeruginosa* PA14 *ΔmotCD fliC*^T394C^	This report
SMC9328	*P. aeruginosa* PA14 *ΔmotAB fliC*^*T394C*^	This report
SMC7581	*P. aeruginosa* PA14 *ΔflgK fliC*^T394C^	This report
SMC241	*P. aeruginosa* PA14/pSMC21 (Kan-r)	Bloemberg 1997 (56)
SMC2386	*P. aeruginosa* PA14 *ΔmotCD*/pSMC21 (Kan-r)	This study
SMC2387	*P. aeruginosa* PA14 *ΔmotAB*/pSMC21 (Kan-r)	This study
SMC3501	*P. aeruginosa* PA14/pMQ72 (Gent-r)	Shanks, AEM
SMC7659	*P. aeruginosa* PA14 *ΔmotCD*/pMQ72 (Gent-r)	Baker 2019 (15)
SMC6698	*P. aeruginosa* PA14 *ΔmotAB*/pMQ72 (Gent-r)	This report
SMC4638	*P. aeruginosa* PA14 *motA*::His_6_	Kuchma 2015 (23)
SMC9206	*P. aeruginosa* PA14 *motC*::His_6_	This report
SMC9207	*P. aeruginosa* PA14 *motA*::His_*6*_ *motC*::His_6_	This report
SMC5790	*P. aeruginosa* PA14 *ΔmotC*/pMQ72	Kuchma 2015 (23)
SMC5791	*P. aeruginosa* PA14 *ΔmotC*/pMotC::His_6_ (Gent-r)	Kuchma 2015 (23)
AB327	*P. aeruginosa* PA14/pMotCD (Gent-r)	This report
AB325	*P. aeruginosa* PA14 *ΔmotCD*/pMotCD(Gent-r)	This report
SMC7229	*P. aeruginosa* PA14 *ΔmotAB*/pMotAB (Gent-r)	This report

### Strain construction

In-frame insertion of the His_6_ epitope tag into the *motA* and *motC* genes was performed via allelic exchange, as previously described (57). Plasmids for this purpose were constructed via cloning by homologous recombination of relevant PCR products into the pMQ30 vector using Gibson assembly. Constructs for plasmid-based expression of genes were generated using PCR and Gibson Assembly (NEB, Boston, MA) followed by cloning into pMQ72. For all plasmids and constructs used in the experiments described herein, the relevant cloned genes were fully sequenced to confirm that the correct sequences were present. PCR and sequencing were also used to confirm the presence of the His_6_ epitope tag in the *motC* gene on the chromosome in the *motA*::His_6_ *motC*::His_6_ and *motC*::His_6_ strains.

### Protein detection and cellular localization experiments

Bacterial strains were grown either in liquid cultures or on swarm agar plates in M63 minimal salts medium supplemented with 1 mM MgSO_4_, 0.2% glucose, and 0.5% casamino acids (CAA) with 0.5% arabinose for induction of plasmid-based expression of the pMotC::His_6_ protein. Whole cell lysates and membrane fractions were prepared as previously described (58). Total protein concentrations in membrane fractions were quantified using the Pierce BCA protein assay kit (Thermo Fisher Scientific, Waltham, MA). For Western blotting, equivalent total protein quantities from membrane samples were resolved by SDS-PAGE using Any kD polyacrylamide gels (Bio-Rad, Hercules, CA). Proteins transferred to a nitrocellulose membrane were probed with a monoclonal anti-His_6_ antibody (Qiagen, Germantown, MD). Detection of proteins via Western blotting was performed by fluorescence detection using IR-Dye-labeled fluorescent secondary antibodies and imaged using the Odyssey CLx Imager (LICOR Biosciences, Inc., Lincoln, NE). Protein quantification was performed using Image Studio Lite software (LICOR Biosciences, Inc., Lincoln, NE).

### Motility assays

Swarm motility plates were prepared with M63 medium supplemented with 1 mM MgSO_4_, 0.2% glucose, and 0.5% CAA, referred to as M63 medium in this report, and solidified with 0.5% agar. Swarm assays were performed as previously described (59). Swim motility plates were prepared with M63 medium supplemented with 1 mM MgSO_4_, 0.2% glucose, and 0.5% CAA and solidified with 0.3% agar. Swim assays were performed as previously described (60). Arabinose was supplemented to media at different concentrations as indicated for strains carrying the arabinose-dependent plasmid.

### Single-cell swimming motility tracking

Cell tracking was performed as previously described (34) with a few minor adaptations. *P. aeruginosa* PA14 strains were incubated in liquid Miller’s Luria-Bertani broth (LB) medium with shaking at 37°C overnight. Cells were washed with M63 medium; for the strains carrying an arabinose-inducible plasmid pMQ72, the M63 medium was supplemented with 1% arabinose. The washed culture was diluted to OD_600_ ~1.0; 20 µL of this culture was inoculated into 1 mL of M63 medium at different viscosities and incubated for 1.5 hours without shaking at 37°C before imaging. Methylcellulose 400 cP powder (Sigma-Aldrich) at varying percent concentrations was used to change the viscosity of the M63 medium following the manufacturer’s directions for methylcellulose hydration. Glucose (0.2%), MgSO_4_ (1 mM), and casamino acids (0.5%) were added after hydration.

The cells were injected into a flow cell channel (Ibidi sticky-Slide VI0.4 with a glass coverslip). Bright-field imaging recordings were taken using a Phantom V12.1 high-speed camera (Vision Research) with a 5-ms exposure at 200 frames per second (fps) and a 0.1 µm/pixel resolution on 600 × 800 pixels field of view. The imaging protocol was performed on an Olympus IX83 inverted microscope equipped with a 100× oil objective, a 2× multiplier lens, and Zero Drift Correction autofocus system and a heating stage (30°C).

Image processing and cell tracking of the near-surface swimming recordings were processed with algorithms written in MATLAB R2015a (MathWorks) described in previous work (34). Swimming trajectories were identified as those that traced a radius of gyration ≥2.5 µm and a ballistic movement with MSD slope ≥1.4; these thresholds discriminated against too short and/or passive (diffusive) trajectories. The reported speeds per trajectory are the calculated median speed from a distribution displacements using 20 frames (100 ms) moving window over the full trajectory.

### Agar-liquid film cultures

To mimic the swarming lag phase of a swarm plate assay, warm 0.55% agar M63 medium was poured onto a 25 × 25 × 2.5 mm silicone CoverWell imaging chamber (GraceBio) with a bottom glass coverslip. Immediately after, a glass coverslip was laid to create a flat surface. After 1 hour, the top coverslip was removed, leaving a flat soft agar surface. The surface was allowed to dry for ~20 min until a slight meniscus is formed. Ten microliters of washed and diluted (to OD_600_ ~1.0) overnight culture were inoculated onto the agar surface, as described above. A clean glass coverslip was laid on top of the inoculated culture to create a thin film of liquid culture sealed between the agar and the glass imaging surface. The sealed imaging chambers were incubated at 37°C and only taken out of the incubator for imaging, ~10 min, and then returned to incubator. Imaging and data processing were performed as above for single-cell swimming motility tracking. Trajectories were classified as follows: flagellar motile, a ballistic movement with MSD slope ≥1.4, or flagellar immotile, a diffusive movement with MSD slope ≥0.7, but ≤1.3. Cells with MSD slope <0.7 were considered surface attached. For flagellar motile trajectories, the reported speeds per trajectory are the calculated median speed from a distribution displacements using 20 frames (100 ms) moving window over the full trajectory.

### Crowded environment assays

A 25 × 25 × 2.5 mm silicone CoverWell imaging chamber (GraceBio) with a bottom glass coverslip was filled with heated M63 medium containing 0.55% agar, and a glass coverslip was pressed on top, as above. The top coverslip was removed, and excess liquid was allowed to dry for ~5 min. To inoculate the miniature plates, cells were harvested from a swarming motility plate after 10 hours of incubation. Using a toothpick to collect a sample from the tip of a tendril or edge of the colony for swarming-deficient strains, the sampled biomass was placed at the center of the agar surface on the miniature plate. A clean imaging coverslip was gently pressed on the top to firmly enclose the cells against the agar and conserve humidity; smearing or distortion of the inoculum from the center was carefully avoided. The miniature plates were incubated at 37°C for 3 hours. After incubation period, the cells traveled radially outward from the center inoculum and imaged.

To track individual cells in the expanding crowded environments, a fraction of cells constitutively expressing a GFP-carrying plasmid, pSMC21, were co-inoculated in the source swarming motility plate. The strains with and without the GFP reporter were mixed to a 1:99 volume ratio, respectively, from separate washed and diluted (OD_600_ ~1.0) overnight cultures. The co-culture was inoculated to a swarming motility plate and incubated as before. The plate was sampled after 10 hours of incubation as described above. A higher volume ratio of 5:95 was used for swarming-deficient strains due to their lower collective motility in the confined crowded environment which reduces their level of mixing as the crowd expands during incubation in the miniature agar plate, i.e., only the ΔMotCD pSMC21 cells closest to the edge of the new sampled inoculum will be carried by the expanding front. No significant changes in swarming phenotype were observed for the strains carrying the plasmid pSMC21 (Fig. S7) compared to the background strain.

Imaging recordings were taken with an Andor Neo sCMOS camera with Andor IQ software on an Olympus IX83 microscope equipped with a 100× oil objective and Zero Drift Correction continuous autofocus system. To avoid bias of the constrained edge cells, the XYZ location for the recording was set ~15 µm from the edge of the expanding crowded environment. One-minute fluorescence recordings were taken with 100-ms exposure for a 10-fps recording (shutters were continuously open and without display feedback to maximize frame rate) with Lambda LS (Sutter Instrument) xenon arc lamp and a GFP filter. The image size was 133 µm by 133 µm (2,048 by 2,048 pixels). Except for the Δ*motCD* strain, all other strains were imaged with this protocol. We noticed that with a continuously open shutter protocol, the ΔmotCD strain progressively reduced displacement. We believe that this could be attributed to the low level of mixing in the crowd, i.e., individual cells cannot move away from the fluorescent focal spot to reduce their accumulated fluorescence exposure time, as compared to the other motor strains. Reduction of the frame rate to 1 fps with an active open-close shutter solved this issue by reducing the accumulated exposure in the Δ*motCD* strain without compromising resolution, since the strain moves slower in this crowded regime relative to the other motor strains. Image processing and cell tracking of the GFP fluorescent cells were processed with algorithms written in MATLAB R2015a (Mathworks) as described above.

Individual flagellum tracking was performed similarly as above. A *P. aeruginosa* PA14 strain with a FliC protein modified, a threonine-to-cystine mutation, FliC^T394C^, as previously described (34), was co-inoculated with a strain not carrying this mutation. The strains with and without the FliC^T394C^ mutation were mixed to a 5:95 volume ratio, respectively, from separate washed and diluted (OD_600_ ~1.0) overnight cultures; similar to the single-cell tracking in a crowded environment described above, higher volume ratio of 1:4 was used for swarming-deficient strains. The mixed culture was inoculated to a swarming motility plate and incubated as before. After incubation, the flagella were stained using Alexa Fluor 488 C5 Maleimide (Molecular Probes) at 10 µg/mL. The edge of a tendril or colony was stained with ~1 µL of the stock stain and allowed to sit for 10 min before the biomass was sampled for imaging. Imaging protocol was the same as described above. Only intensity rescaling was used on the raw fluorescent images. Quantification of active and inactive flagellum was done manually using MATLAB R2015a to display the recording. Each flagellum in the field of view was assessed for activity by playing the recordings multiple times by two independent observers. Flagella were considered to be active if they performed one or more of the following criteria during the recording period: (i) the filament performed a standing wave, i.e., rotation of extended filament (34; (ii) filament transitioned between body-wrapped and extended states (61); or (iii) filament performed rotation in the body-wrapped state (61). The fraction of inactive flagella, therefore, is one minus the fraction of active flagella.

### Free-swimming on 2D-agar surface

A miniature soft agar plate was prepared as described above. After removal of top glass coverslip, 2 µL of M63 media was placed on the agar surface. Cells were sampled from a standard swarm plate using a plastic inoculation loop. The loop was smeared on a fresh swarming motility plate to dilute the sample. The diluted sample on the inoculation loop was gently caressed over a miniature agar plate with liquid medium. An imaging glass coverslip was gently pressed to seal the silicone agar surface for imaging. Only flat areas where cells are visibly swimming in a constrained 2D space across the field of view with minimal out-of-plane body rotation were imaged (Movie S4). We estimated 2D liquid volume between agar surface and glass coverslip to be 1–2 μm due to the visible restriction on out-of-focal plane body rotation, “wobbling,” while still maintaining swimming motility. Bright-field imaging using a high-speed camera (Vision Research), image processing, and single-cell tracking were performed as described above.

### Mixed population swarming assays

Swarming assays of mixed swarming-deficient strains (*ΔflgK*—lacking a flagellum) and the different swarming strains were performed as described above, with some modifications. Overnight cultures of the selected strains were washed using M63 medium and then normalized to an OD_600_ of ~1.0. The washed cultures were then mixed to the desired testing ratios to a final volume of 100 µL. Finally, 2 µL of the co-culture mixture was inoculated to a 0.55% agar swarm plate assay as described before and allowed to grow for 18 hours at 37°C. Plates were not stacked to avoid temperature gradients on the plates.

### Statistical analysis

One-way analysis of variance with multiple comparisons was performed pairwise between all isolates using the GraphPad Prism 6 software or Matlab software.

## SUPPLEMENTAL MATERIAL

Figure S1 mbio.03322-23-s0001.tiff Expression of the His-tagged stators proteins can promote WT levels of both swimming and swarming motility when expressed at their endogenous locus.

Figure S2 mbio.03322-23-s0002.tiff Compiled expression profiling of *motA*, *motB*, *motD*, *flhA*, *flhF*, *fleN*, *fliA*, *cheY*, *cheZ*, *cheA* and *cheB* levels relative to *motC* levels in *Pseudomonas aeruginosa* from five separate

Figure S3 mbio.03322-23-s0003.tiff Calculated viscosity values for swarming lag liquid environment.

Figure S4 mbio.03322-23-s0004.tiff Modulation of viscosity parameter, η_s_, in equations (4) and (7) in of the translational and rotational friction coefficients.

Figure S5 mbio.03322-23-s0005.tiff Measurement of swarming motility and swimming speeds for cells carrying arabinose inducible plasmid at 0% arabinose.

Figure S6 mbio.03322-23-s0006.tiff Measurement of swimming speeds for cells carrying arabinos inducible plasmid at 1% arabinose.

Figure S7 mbio.03322-23-s0007.tiff Swarming motility of WT and stator mutants with and without *fliC*^T394C^ or constitutive plasmid pSMC21.

Movie S1 mbio.03322-23-s0008.mp4 Sample movie of ΔMotAB FliC^T394C^ stained flagella in a crowded environment to quantify flagellum activity.

Movie S2 mbio.03322-23-s0009.mp4 Sample movie of ΔMotCD FliC^T394C^ stained flagella in a crowded environment to quantify flagellum activity.

Movie S3 mbio.03322-23-s0010.mp4 Sample movie of WT FliC^T394C^ stained flagella in a crowded environment to quantify flagellum activity.

Movie S4 mbio.03322-23-s0011.mp4 Sample movie of swimming cells in a constrained 2D-space, between imaging glass coverslip and 0.55% soft agar with nutrients.

Movie S5 mbio.03322-23-s0012.mp4 Movie of crowd simulation at a 0.9 fraction of motile cells with *F_f_* of 0.02.

Movie S6 mbio.03322-23-s0013.mp4 Movie of crowd simulation at a 0.7 fraction of motile cells with *F_f_* of 0.02.

Movie S7 mbio.03322-23-s0014.mp4 Movie of crowd simulation at a 0.4 fraction of motile cells with *F_f_* of 0.02.

Movie S8 mbio.03322-23-s0015.mp4 Movie of crowd simulation at a 0.1 fraction of motile cells with *F_f_* of 0.02.

Supplemental methods mbio.03322-23-s0016.pdf Supplemental methods for crowded simulations.

Supplemental legends mbio.03322-23-s0017.pdf Legends for supplemental figures and movies.

ASM does not own the copyrights to Supplemental Material that may be linked to, or accessed through, an article. The authors have granted ASM a non-exclusive, world-wide license to publish the Supplemental Material files. Please contact the corresponding author directly for reuse.
