# Supplementary figures and images for: How P. aeruginosa cells with diverse stator composition collectively swarm
Source: mBio. 2024 Mar 1;15(4):e03322-23. doi: 10.1128/mbio.03322-23 (PMC11005332; doi:10.1128/mbio.03322-23)

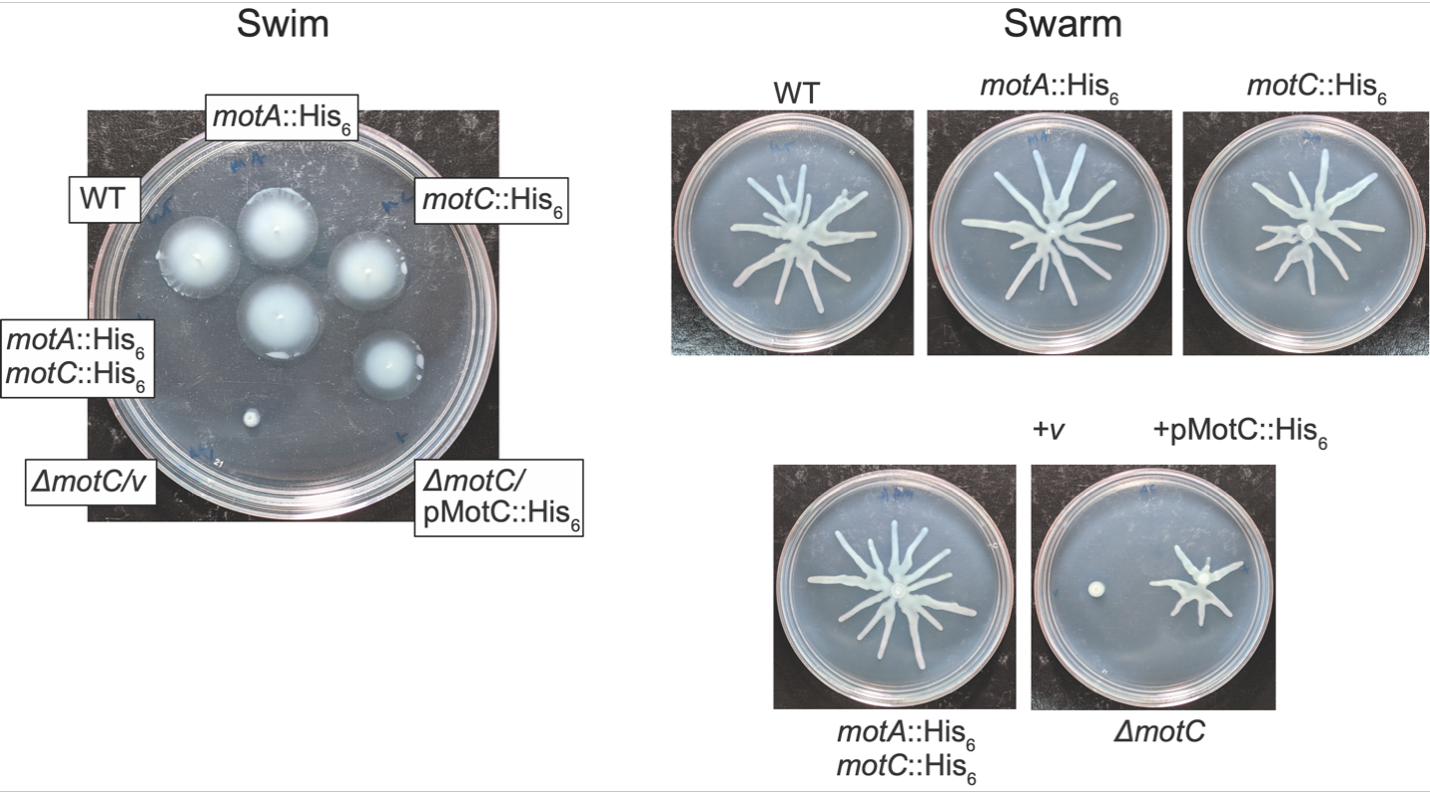

Supplement: Figure S1 — Expression of the His-tagged stators proteins can promote WT levels of both swimming and swarming motility when expressed at their endogenous locus. [file mbio.03322-23-s0001.tiff]

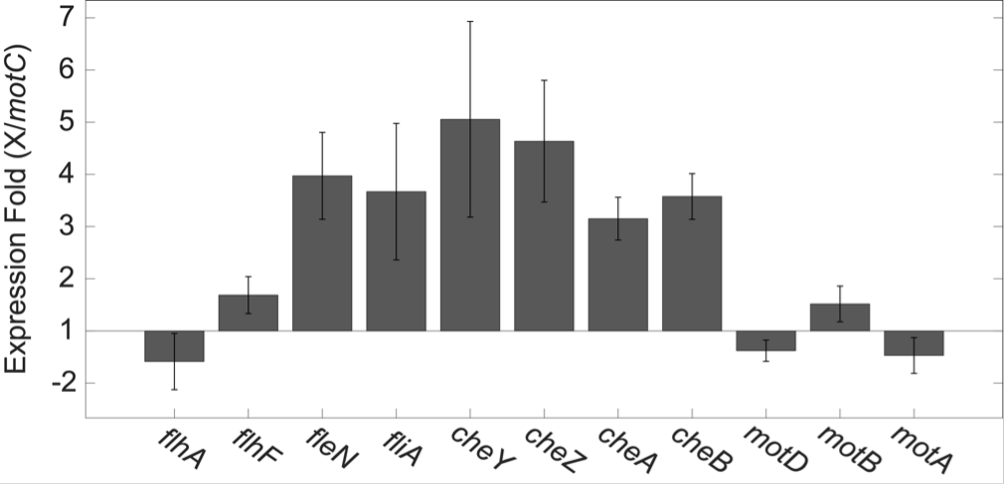

Supplement: Figure S2 — Compiled expression profiling of motA, motB, motD, flhA, flhF, fleN, fliA, cheY, cheZ, cheA and cheB levels relative to motC levels in Pseudomonas aeruginosa from five separate [file mbio.03322-23-s0002.tiff]

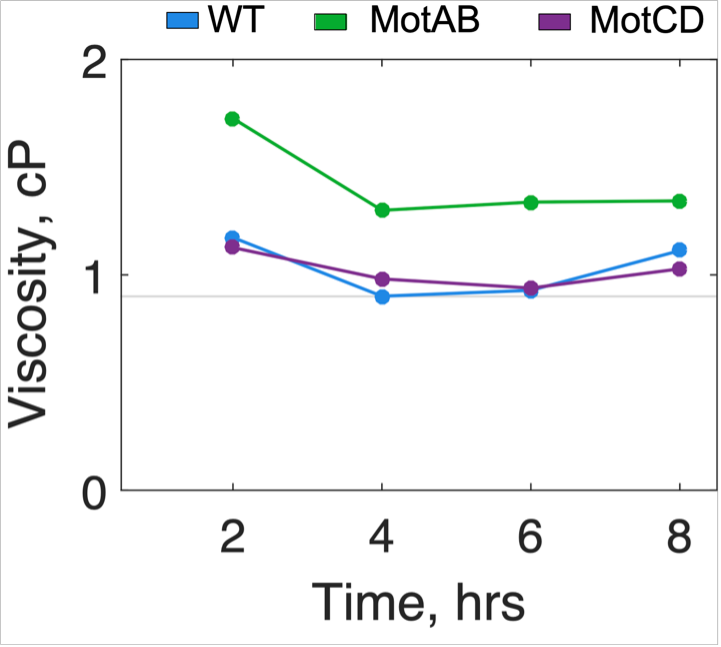

Supplement: Figure S3 — Calculated viscosity values for swarming lag liquid environment. [file mbio.03322-23-s0003.tiff]

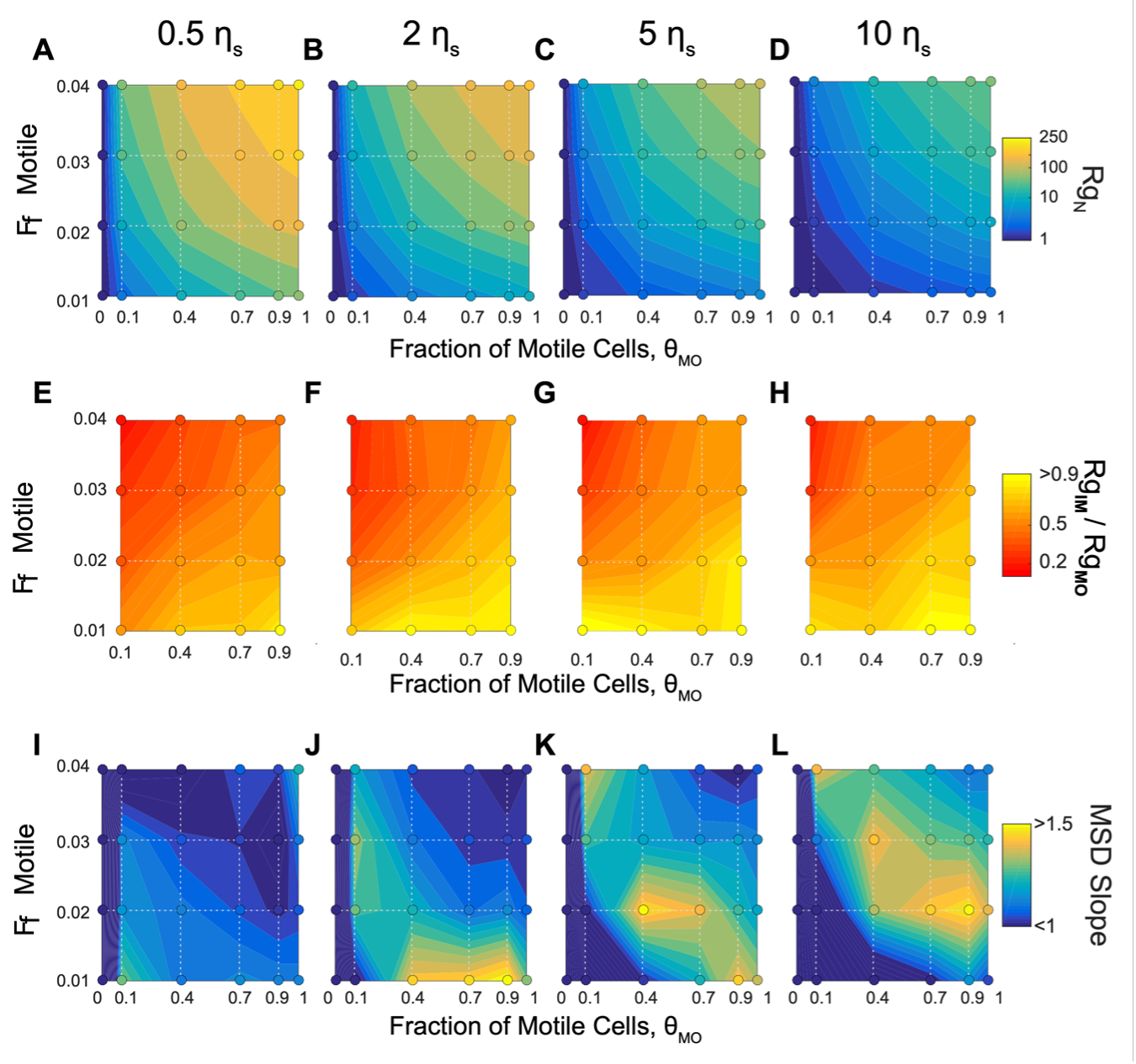

Supplement: Figure S4 — Modulation of viscosity parameter, ηs, in equations (4) and (7) in of the translational and rotational friction coefficients. [file mbio.03322-23-s0004.tiff]

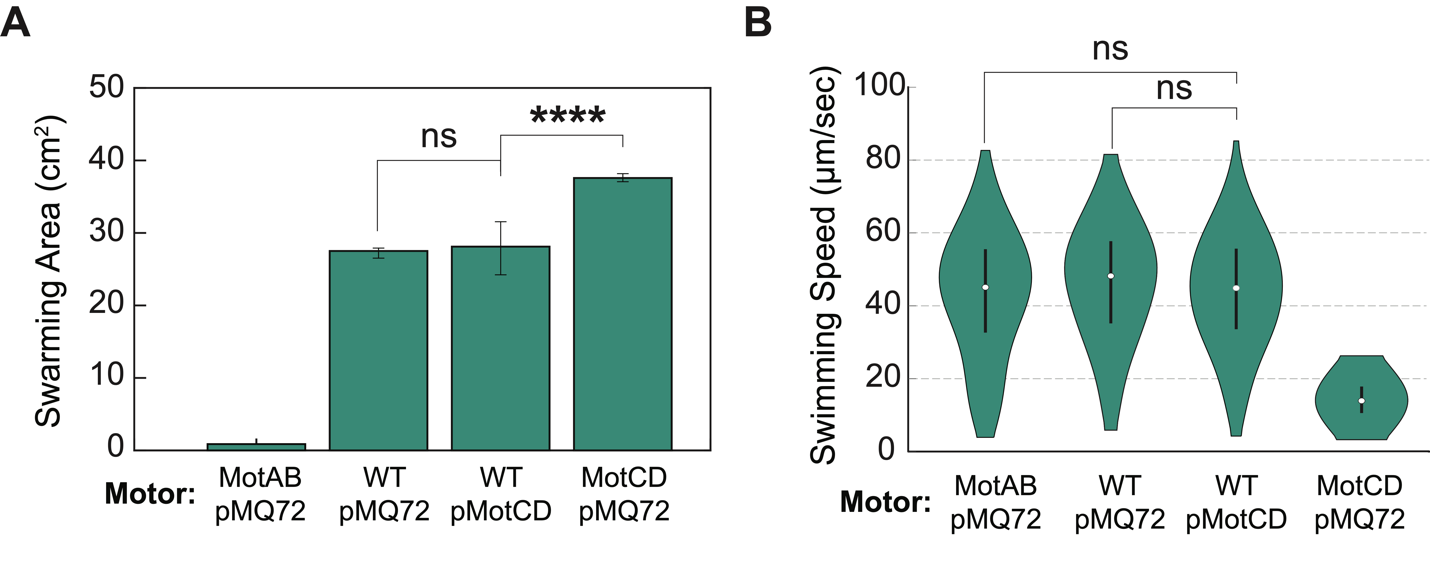

Supplement: Figure S5 — Measurement of swarming motility and swimming speeds for cells carrying arabinose inducible plasmid at 0% arabinose. [file mbio.03322-23-s0005.tiff]

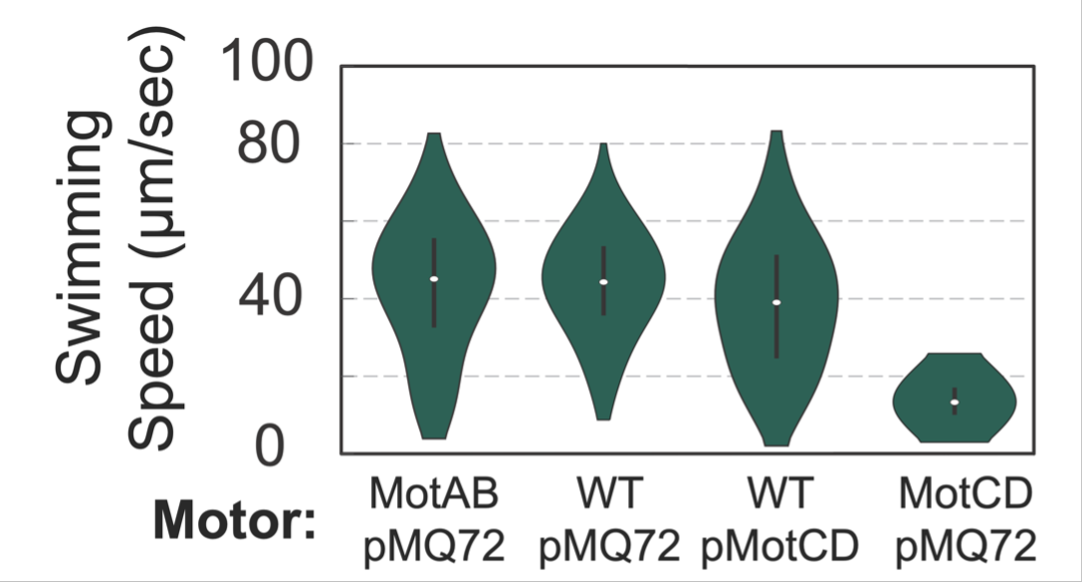

Supplement: Figure S6 — Measurement of swimming speeds for cells carrying arabinos inducible plasmid at 1% arabinose. [file mbio.03322-23-s0006.tiff]

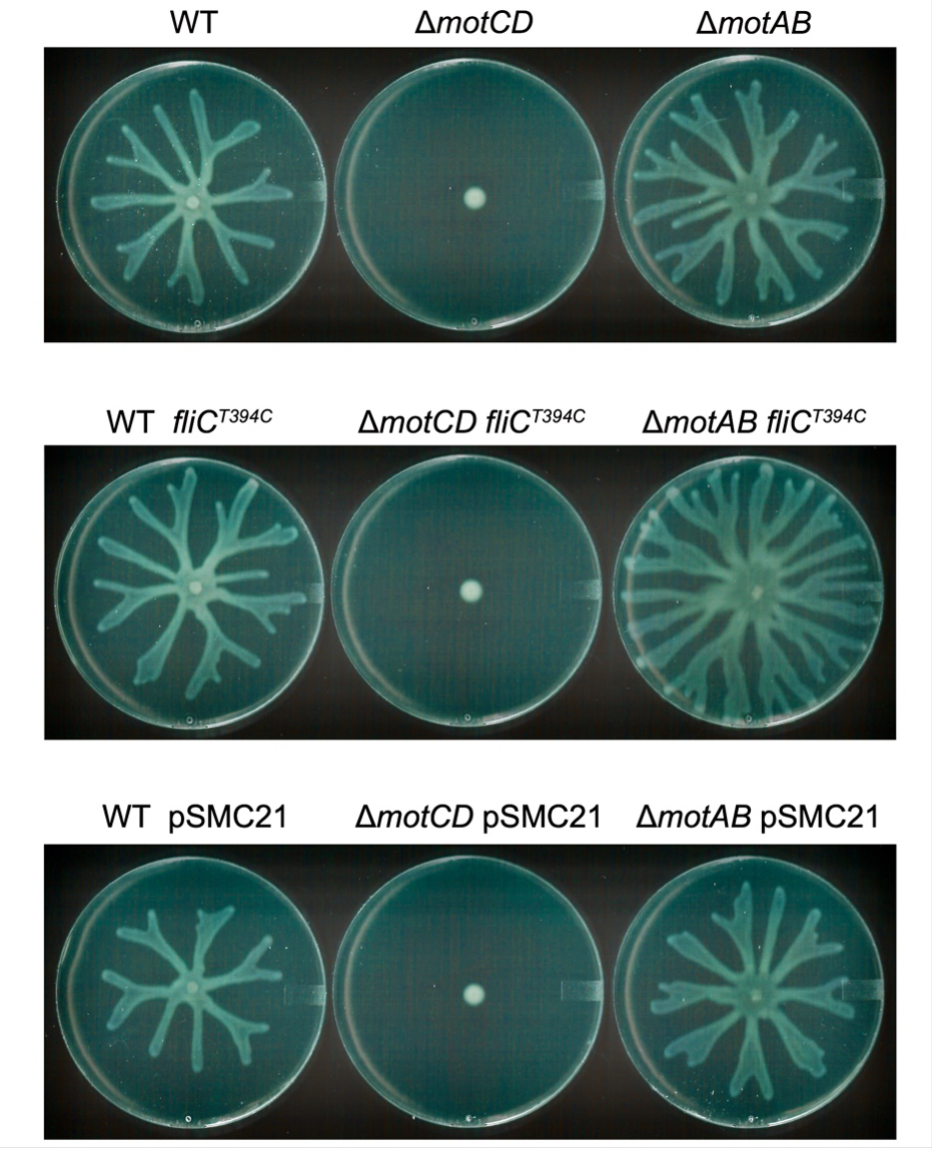

Supplement: Figure S7 — Swarming motility of WT and stator mutants with and without fliCT394C or constitutive plasmid pSMC21. [file mbio.03322-23-s0007.tiff]
