# Supplementary material for: How P. aeruginosa cells with diverse stator composition collectively swarm
Source: mBio. 2024 Mar 1;15(4):e03322-23. doi: 10.1128/mbio.03322-23 (PMC11005332; doi:10.1128/mbio.03322-23)
Supplement: Supplemental methods — for crowded simulations. [file mbio.03322-23-s0016.pdf]

Supplemental Methods

Modeling of cell populations with heterogeneous motor outputs.

Using discrete element method simulations, we study the collective motion of an ensemble of active 2D circulo-lines. The ciculo-line shape accounts for the elongated geometry of bacteria. Each circulo-line i is defined by the endpoints, v_{1i} *and* v_{2i} , length l_i, and thickness, $2\delta_i$. A ciruclo-line is defined as the collection of points that are equidistant from the line connecting the vertices v_{1i} and v_{2i}. The aspect ratio of the circulo-line is $\alpha_i = \frac{l_i}{2\delta_i}$, set to 4 for these simulations. Cicrulo-lines i and j interact via the pairwise, purely repulsive linear spring potential:

$$U(r_{ij}) = \frac{k}{2}\left(\sigma_{ij} - r_{ij}\right)^2 \Theta\left(\sigma_{ij} - r_{ij}\right), \tag{1}$$

where k is the spring constant, $\Theta(.)$ is the Heaviside step function, which prevents interactions between circulo-lines that are not in contact, r_{ij} is the closest distance between interacting circulo-lines, and $\sigma_{ij} - r_{ij}$ is the magnitude of the overlap between the two particles. Following the procedure described previously(1), we ensure that the potential energy and forces, $\vec{F_i} = \vec{\nabla}_i U$, are continuous when the circulo-lines come in and out of contact.

We initialize the simulations by placing 324 ciruclo-lines with random positions and orientations in a square box with side length L = 1, periodic boundary conditions, and

low packing fraction φ=$\frac{\sum_{i=1}^{N}(\pi\delta_i{}^2+2l_i\delta_i)}{L^2}=0.2$. We quasistatically compress the system in small steps $\Delta\phi$ until we reach the final packing fraction φ_0=0.96.

After reaching $\varphi_{0,}$ the position of each particle is updated using Langevin dynamics:

$$m_i\partial_t\boldsymbol{V_i}=-\gamma_{\parallel}V_{\parallel i}\widehat{\boldsymbol{u}}_{\parallel}-\gamma_{\perp}V_{\perp i}\widehat{\boldsymbol{u}}_{\perp}-\sum_{i\neq j}\boldsymbol{\nabla}U_{ij}+\boldsymbol{F}_f+\sqrt{2D}\boldsymbol{\xi}^T(t), \tag{2}$$

where $\boldsymbol{V_i},\boldsymbol{F_f},$ and $\xi(t)$ are the velocity of each circulo-line, active force, and Gaussian white noise with < $\xi^T(t)$>=0 and <$\xi^T(t)\xi^T(t') >= 2D_T\delta(t-t')$, with D_T being the translational diffusion coefficient, respectively. $\widehat{\boldsymbol{u}}_{\parallel}$ is defined from the tail to the head of the of circulo-line, i.e., $\frac{v_{2i}-v_{1i}}{|v_{2i}-v_{1i}|}$, and $\widehat{\boldsymbol{u}}_{\perp}$ is the unit vector normal to $\widehat{\boldsymbol{u}}_{\parallel}$.

Each simulation contained a fixed fraction of active ($\boldsymbol{F_f}$>0) and inactive bacteria ($\boldsymbol{F_f}$=0), see Movies S5-8. The relative magnitude of the active force is measured compared to the repulsive interaction coefficient, k in equation (1). The flagellar motor is usually not uniformly pushing in one direction over long times, it may perform temporary stochastic events of motor halt or directionality reversal (2, 3). Hence, to design more biologically relevant model we introduced stochasticity to the system by randomly selecting 0.1 fraction of the flagellum active particles ($\boldsymbol{F_f}$>0) to halt, and another 0.1 fraction to reverse direction of $\boldsymbol{F_f}$. The states are updated every 4x10^{-4} τ_D for 12 τ_D where $\tau_D\sim\frac{l_i^2}{D_T}$ is measured in the low-density limit. Finally, flagella are not always aligned with the cell body (2), hence the active force's direction is defined as:

$$\boldsymbol{F_f} = F_f sin(\beta)\hat{\boldsymbol{u}}_{\parallel} + F_f cos(\beta)\hat{\boldsymbol{u}}_{\perp} \tag{3}$$

where were β is random angle between the cell body axis and the flagella as defined previously (2) with centered at $\pi/2$ and standard deviation $\pi/8$, restricted to values between 0 and π.

The translational friction coefficients acting on each ciculo-line are calculated as described in (4):

$$\gamma_{\parallel} = \frac{2\pi\eta_s l_i}{\ln{(a_i)}} \tag{4}$$

$$\gamma_{\perp} = 2\gamma_{\parallel} \tag{5}$$

were η_s denotes the viscosity of the solvent.

We also consider the rotational dynamics for each circulo-line following the Langevin equation:

$$I_i \partial_t \boldsymbol{\omega_i} = -\gamma_r \boldsymbol{\omega}_i + \boldsymbol{T_B}, \tag{6}$$

where $I, \boldsymbol{\omega}, \gamma_r, \boldsymbol{T}_B$, are moment of inertia, angular velocity, rotational friction coefficient, and torque from interaction with other circulo-lines. The rotational friction coefficients acting on each ciculo-line are calculated as described in (4):

$$\gamma_r = \frac{\pi \eta_s l_i^3}{3 \ln (a_i)} \quad (7)$$

The values for the rotational and translational damping coefficients are set according to equation (2). The mean square displacement (MSD) for time ~ 5 τ_D was calculated as follows:

$$MSD = \frac{1}{N} \sum_{i=1}^{N} |\boldsymbol{r}_i(t) - r_i(t=0)|^2 \quad (8)$$

References

1. K. VanderWerf, W. Jin, M. D. Shattuck, C. S. O'Hern, Hypostatic jammed packings of frictionless nonspherical particles. *Phys Rev E* **97**, 012909 (2018).
2. J. de Anda *et al.*, High-speed “4D” computational microscopy of bacterial surface motility. *ACS Nano* **11**, 9340-9351 (2017).
3. Z. Wu, M. Tian, R. Zhang, J. Yuan, Dynamics of the two stator systems in the flagellar motor of *Pseudomonas aeruginosa* studied by a bead assay. *Applied and Environmental Microbiology* **87**, e01674-01621 (2021).
4. Y.-G. Tao, W. K. d. Otter, J. T. Padding, J. K. G. Dhont, W. J. Briels, Brownian dynamics simulations of the self- and collective rotational diffusion coefficients of rigid long thin rods. *The Journal of Chemical Physics* **122**, 244903 (2005).
