# Supplementary material for: How P. aeruginosa cells with diverse stator composition collectively swarm
Source: mBio. 2024 Mar 1;15(4):e03322-23. doi: 10.1128/mbio.03322-23 (PMC11005332; doi:10.1128/mbio.03322-23)
Supplement: Supplemental legends — Legends for supplemental figures and movies. [file mbio.03322-23-s0017.pdf]

Supplemental Figures and Movies Legends

Figure S1. Expression of the His-tagged stators proteins can promote WT levels of both swimming and swarming motility when expressed at their endogenous locus. Left panel shows a representative image of a swim motility assay of the indicated strains. Strains were grown for 16h on soft agar (0.3%) plates with M63 minimal salts medium supplemented with glucose, MgSO4, and CAA. Note: No arabinose was added under these conditions. The basal levels of expression of MotC::His_6 expressed from the plasmid is sufficient for complementation of the non-motile Δ*motC* strain. The right panel shows swarm motility plates of the indicated strains grown for 16h in swarm agar (0.5%) plates with M63 minimal salts medium supplemented with glucose, MgSO4, and CAA. Note: Arabinose was added to 0.5% to facilitate complementation of the non-motile Δ*motC* strain with pMotC::His_6.

Figure S2. Calculated viscosity values for *swarming lag* liquid environment. Viscosity values calculated from measured mean squared displacement (MSD) slopes of diffusive cells, OFF, for experimental set up of Fig. 2A, the *swarming lag* phase microenvironment. Gray line indicates a viscosity of 0.91 cP for water at 25°C.

Figure S3. Modulation of viscosity parameter, $\boldsymbol{\eta_s}$, in equations (4) and (7) of the translational and rotational friction coefficients. The viscosity that each particle feels due to the solvent was varied as 0.5, 2, 5 and 10 η_s for 324 particles at packing fraction of φ_0=0.96. (A-D) The normalized mean radius of gyration, Rg_N (normalized by the mean radius of gyration of the immotile θ_{MO} = 0 system) for all particles in each F_f and θ_{MO} system configuration. (E-H) The asymmetry in translational movement between the motile and immotile fractions for the heterogenous systems were measured as the ratio in mean Rg for the two populations (Rg_{IM}/Rg_{MO}). A Rg_{IM}/Rg_{MO} < 1 corresponds to longer trajectories performed by the motile fraction, relative to the motility that the motile fraction induced on the immotile fraction in the crowd; a ratio of 1 corresponds to equal degree of translation performed by both immotile and motile particles. (I-L) MSD slopes of log-log fits at the late 0.1 fraction of lag time for each system. Contour map was estimated by interpolation between the grid of tested conditions (circular markers).

Figure S4. Swarming motility of WT and stator mutants with and without *fliC*^{T394C} or constitutive plasmid pSMC21. Swarm motility plates of the indicated strains grown for 16h in swarm agar (0.55%) plates with M63 minimal salts medium supplemented with glucose, MgSO4, and CAA. All *ΔmotCD* strains remained swarming deficient, while the *ΔmotAB* strains displayed enhanced swarming motility compared to their respective WT counterparts. The plates were scanned with a HP photosmart 6520 all-in-one scanner. The presented images were post-edited for visualization with color and light enhancement.

Figure S5. Measurement of swarming motility and swimming speeds for cells carrying arabinose inducible plasmid at 0% arabinose. *(A)* At 0% arabinose, MotCD carried on an arabinose-inducible plasmid (pMotCD) does not increase WT swarming. Error bars denote the first and third quartiles of the distribution about the mean. At least

6 plate replicates per condition. *(B)* Expression of MotCD under the control of arabinose in WT does not significantly affect swimming speeds at 0% arabinose induction. At least 300 trajectories per distribution. ****$P < 0.00001$; ns, not significantly different. Data were analysed by one-way ANOVA followed by Tukey's post-test comparison. ns, not significantly different.

Figure S6. Measurement of swimming speeds for cells carrying arabinos inducible plasmid at 1% arabinose. Expression of MotCD via an arabinose-inducible plasmid in the WT does not lead to altered swimming speed at 1% arabinose induction. At least 300 trajectories per distribution.

Figure S7. Compiled expression profiling of *motA, motB, motD, flhA, flhF, fleN, fliA, cheY, cheZ, cheA and cheB* levels relative to *motC* levels in *Pseudomonas aeruginosa* from five separate studies. Genome expression profiles from five separate studies on liquid culture grown *Pseudomonas aeruginosa* were compiled (1-5). Four data sets were extracted from Gene Expression Omnibus (GEO) repository (GEO accessions: GSE10030, GSE6741, GSE4614, GSE3090); and one profile was available as supplementary information to the manuscript (5). The expression levels for each gene were normalized relative to *motC* expression for each study before they were compiled together. Error bars denote the standard deviation on relative expression for all five studies.

Movie S1. Sample movie of ΔMotAB $FliC^{T394C}$ stained flagella in a crowded environment to quantify flagellum activity. Only flagella with a standing wave motion, translational movement or cell body wrapping were considered active. The inoculation volume ratio between strains was ΔMotAB:ΔMotAB $FliC^{T394C}$ = 95:5.

Movie S2. Sample movie of ΔMotCD $FliC^{T394C}$ stained flagella in a crowded environment to quantify flagellum activity. Only flagella with a standing wave motion, translational movement or cell body wrapping were considered active. The inoculation volume ratio between strains was ΔMotCD:ΔMotCD $FliC^{T394C}$ = 4:1.

Movie S3. Sample movie of WT $FliC^{T394C}$ stained flagella in a crowded environment to quantify flagellum activity. Only flagella with a standing wave motion, translational movement or cell body wrapping were considered active. The inoculation volume ratio between strains was WT:WT $FliC^{T394C}$ = 95:5.

Movie S4. Sample movie of swimming cells in a constrained 2D-space, between imaging glass coverslip and 0.55% soft-agar with nutrients. Cells were collected from the edge of WT swarm after 16 hours of growth for imaging. The 1 second of trailing trajectory is drawn for motile cells with a radius of gyration greater than 5μm.

Movie S5. Movie of crowd simulation at a 0.9 fraction of motile cells with F_f of 0.02. There are 324 cells in the crowd with an aspect ratio of 4 and constrained to a 0.96 volume fraction at $10\eta_s$. Rods represent center active (green) and inactive (blue) simulation particles.

Movie S6. Movie of crowd simulation at a 0.7 fraction of motile cells with F_f of 0.02. There are 324 cells in the crowd with an aspect ratio of 4 and constrained to a 0.96 volume fraction at $10\eta_s$. Rods represent center active (green) and inactive (blue) simulation particles.

Movie S7. Movie of crowd simulation at a 0.4 fraction of motile cells with F_f of 0.02. There are 324 cells in the crowd with an aspect ratio of 4 and constrained to a 0.96 volume fraction at $10\eta_s$. Rods represent center active (green) and inactive (blue) simulation particles.

Movie S8. Movie of crowd simulation at a 0.1 fraction of motile cells with F_f of 0.02. There are 324 cells in the crowd with an aspect ratio of 4 and constrained to a 0.96 volume fraction at $10\eta_s$. Rods represent center active (green) and inactive (blue) simulation particles.

References

1. G. G. Anderson, S. Moreau-Marquis, B. A. Stanton, G. A. O'Toole, In vitro analysis of tobramycin-treated *Pseudomonas aeruginosa* biofilms on cystic fibrosis-derived airway epithelial cells. *Infect Immun* **76**, 1423-1433 (2008).
2. C. Alvarez-Ortega, C. S. Harwood, Responses of *Pseudomonas aeruginosa* to low oxygen indicate that growth in the cystic fibrosis lung is by aerobic respiration. *Mol Microbiol* **65**, 153-165 (2007).
3. S. Chugani, E. P. Greenberg, The influence of human respiratory epithelia on *Pseudomonas aeruginosa* gene expression. *Microb Pathog* **42**, 29-35 (2007).
4. W. Chang, D. A. Small, F. Toghrol, W. E. Bentley, Microarray analysis of *Pseudomonas aeruginosa* reveals induction of pyocin genes in response to hydrogen peroxide. *BMC Genomics* **6**, 115 (2005).
5. N. Dasgupta *et al.*, A four-tiered transcriptional regulatory circuit controls flagellar biogenesis in *Pseudomonas aeruginosa*. *Mol Microbiol* **50**, 809-824 (2003).
